# Sequence variation between 462 human individuals fine-tunes functional sites of RNA processing

**DOI:** 10.1038/srep32406

**Published:** 2016-09-12

**Authors:** Pedro G. Ferreira, Martin Oti, Matthias Barann, Thomas Wieland, Suzana Ezquina, Marc R. Friedländer, Manuel A. Rivas, Anna Esteve-Codina, The GEUVADIS Consortium, Xavier Estivill, Roderic Guigó, Emmanouil Dermitzakis, Stylianos Antonarakis, Thomas Meitinger, Tim M Strom, Aarno Palotie, Jean François Deleuze, Ralf Sudbrak, Hans Lerach, Ivo Gut, Ann-Christine Syvänen, Ulf Gyllensten, Stefan Schreiber, Philip Rosenstiel, Han Brunner, Joris Veltman, Peter A.C.T Hoen, Gert Jan van Ommen, Angel Carracedo, Alvis Brazma, Paul Flicek, Anne Cambon-Thomsen, Jonathan Mangion, David Bentley, Ada Hamosh, Philip Rosenstiel, Tim M Strom, Tuuli Lappalainen, Roderic Guigó, Michael Sammeth

**Affiliations:** 1 Bioinformatics and Genomics, Center for Genomic Regulation (CRG), 08003 Barcelona, Catalonia, Spain; 2 Department of Genetic Medicine and Development, University of Geneva Medical School, 1211 Geneva, Switzerland; 3 Instituto de Investigação e Inovação em Saúde, (i3S) Universidade do Porto, 4200-625 Porto, Portugal; 4 Institute of Molecular Pathology and Immunology (IPATIMUP), University of Porto, 4200-625 Porto, Portugal.; 5 Institute of Biophysics Carlos Chagas Filho (IBCCF), Federal University of Rio de Janeiro (UFRJ), 21941-902 Rio de Janeiro, Brazil; 6 Institute of Clinical Molecular Biology, Christians-Albrechts-Universität zu Kiel, 24105 Kiel, Germany; 7 Institute of Human Genetics, Helmholtz Center Munich, 85764 Neuherberg, Germany; 8 Center for Human Genome and Stem-cell research (HUG-CELL), University of São Paulo (USP), 05508090 São Paulo, Brazil; 9 Science for Life Laboratory, Stockholm University, Box 1031, 17121 Solna, Sweden.; 10 Wellcome Trust Centre for Human Genetics, University of Oxford, Oxford OX3 7BN, United Kingdom; 11 Centre Nacional d’Anàlisi Genòmica, 08028 Barcelona, Catalonia, Spain.; 12 Center for Research in Agricultural Genomics (CRAG), Autonome University of Barcelona, 08193 Bellaterra, Catalonia, Spain; 13 Institute of Human Genetics, Technische Universität München, 81675 Munich, Germany; 14 Institute for Genetics and Genomics in Geneva (iGE3), University of Geneva, 1211 Geneva, Switzerland; 15 Swiss Institute of Bioinformatics, 1211 Geneva, Switzerland; 16 Pompeu Fabra University (UPF), 08003 Barcelona, Catalonia, Spain; 17 National Center of Scientific Computing (LNCC), 2233-6000 Petrópolis, Rio de Janeiro, Brazil; 18 Wellcome Trust Sanger Institute, Hinxton Cambridge CB10 1SA, UK; 19 Centre National de la Recherche Génomique, 91030 Evry, France; 20 Max Planck Institute for Molecular Genetics, 14195 Berlin, Germany; 21 Uppsala University, Box 256 751 05 Uppsala, Sweden; 22 Radboud University Nijmegen Medical Centre, 6500 HB Nijmegen, the Netherlands; 23 Leiden University Medical Center, 2333 ZA Leiden, the Netherlands; 24 Universidad de Santiago de Compostela, E-15706 Santiago de Compostela, Spain; 25 European Bioinformatics Institute, EMBL-EBI, Hinxton Cambridge CB10 1SD, UK; 26 Institut National de la Santé et de la Recherche Médicale, 75013 Paris Country, France; 27 Life Technologies, 64293 Darmstadt, Germany; 28 Illumina Cambridge Limited, Fulbourn Cambridge CB21 5XE, UK; 29 Johns Hopkins University School of Medicine, Baltimore MD 21205, USA

## Abstract

Recent advances in the cost-efficiency of sequencing technologies enabled the combined DNA- and RNA-sequencing of human individuals at the population-scale, making genome-wide investigations of the inter-individual genetic impact on gene expression viable. Employing mRNA-sequencing data from the Geuvadis Project and genome sequencing data from the 1000 Genomes Project we show that the computational analysis of DNA sequences around splice sites and poly-A signals is able to explain several observations in the phenotype data. In contrast to widespread assessments of statistically significant associations between DNA polymorphisms and quantitative traits, we developed a computational tool to pinpoint the molecular mechanisms by which genetic markers drive variation in RNA-processing, cataloguing and classifying alleles that change the affinity of core RNA elements to their recognizing factors. The *in silico* models we employ further suggest RNA editing can moonlight as a splicing-modulator, albeit less frequently than genomic sequence diversity. Beyond existing annotations, we demonstrate that the ultra-high resolution of RNA-Seq combined from 462 individuals also provides evidence for thousands of *bona fide* novel elements of RNA processing—alternative splice sites, introns, and cleavage sites—which are often rare and lowly expressed but in other characteristics similar to their annotated counterparts.

In eukaryotes–especially in mammals–functional mRNAs depend crucially on the correct processing of transcribed sequences, governed by (alternative) splicing and 3′ end formation^1^. At the molecular level these reactions rely on the recognition of the corresponding core RNA elements by different factors involved in transcript processing, i.e., components of the splicing machinery (e.g., U1 and U2) that target the splice site sequences in order to remove introns^2^ and polyadenylation signals that correspondingly bind to the Cleavage/Polyadenylation Specificity Factor (CPSF) for initiating the 3′ formation^3^,^4^. In addition to these central elements, modern molecular biology has demonstrated several scenarios of more complex splicing reactions that regulate the correct abundance of alternative gene products, involving accessory proteins, non-coding RNAs and also epigenetic factors. However, these mechanisms follow very cell-type and gene-specific rules that are not applicable in the general case^5^,^6^,^7^,^8^,^9^.

The genomic sequence varies from individual to individual, and already some published case studies show that genetic markers can affect the control of RNA processing^10^,^11^,^12^. Particularly in human, the causal DNA variants of several diseases have been demonstrated to tamper with the control of splicing^13^,^14^,^15^. Traditionally, best practices for carrying out systematic studies on splicing mechanisms involve specifically designed mutagenesis experiments in minigenes^16^,^17^, which despite their evident usefulness, are restricted to a single locus and mutation in each experiment^18^. Predominantly hampered by the lack of availability of genome-wide genotype and phenotype data across a sufficient number of individuals, mechanistic investigations of differences in RNA-processing throughout populations have so far been limited to small numbers of genes and individuals^19^,^20^,^21^,^22^,^23^. However, the advent of high-throughput sequencing technologies also heralded a new generation of population-scale projects that analyse combined DNA and RNA sequencing across multiple individuals. Such studies generally focus on identifying *which* genetic elements are statistically associated with a certain phenotype—usually defined as a quantitative trait locus (QTL) resolved at gene- transcript- or exon-level—rather than building hypotheses about *how* these phenotypic changes are mechanistically projected from the DNA to the RNA molecules^24^,^25^,^26^,^27^,^28^,^29^,^30^,^31^.

In our present work, we employ data from the Geuvadis Project that provides deep RNA-sequencing in lymphoblastoid cell lines (LCLs) collected from 462 individuals of five populations genotyped in the 1000 Genomes Project^32^. The Geuvadis RNA-Seq experiments are described extensively in Lappalainen *et al*.^33^ with a detailed analysis of the technical variation in ‘t Hoen *et al*.^34^. Our main study^33^ already used this data set to map and to characterize regulatory variation, showing by expression QTL (eQTL) analysis that genetic control of gene expression and transcript processing appears largely independent. Here, we drill into the molecular mechanisms of RNA modifications that are modulated by genetic polymorphisms in the sequence motifs of annotated splice donor and splice acceptor sites at the 5′ and 3′ ends of introns^35^, as well as in poly-A signals affecting the 3′ formation of transcripts^36^,^37^. Beyond genotypes, our study also extends to the effect of additional sequence variants in functional elements that are likely due to RNA editing mediated by the adenosine deaminase acting on RNA (ADAR) enzyme, as observed by divergences of the RNA-Seq reads from the corresponding DNA sequencing data. Combining the resolution of sequencing transcriptomes from hundreds of individuals in a population-scale project, we also pinpoint rare and therefore often not annotated transcriptional elements, i.e. splice sites, introns and cleavage sites. Altogether, our studies describe a comprehensive classification and comparison of the different ways in which RNA processing can be affected by these sources of sequence variation and serve as a reference for forthcoming mechanistic studies on RNA regulation by minority alleles.

## Results

### Genomic variants in splice sites can affect the splicing potential positively or negatively

**Figure 1:**
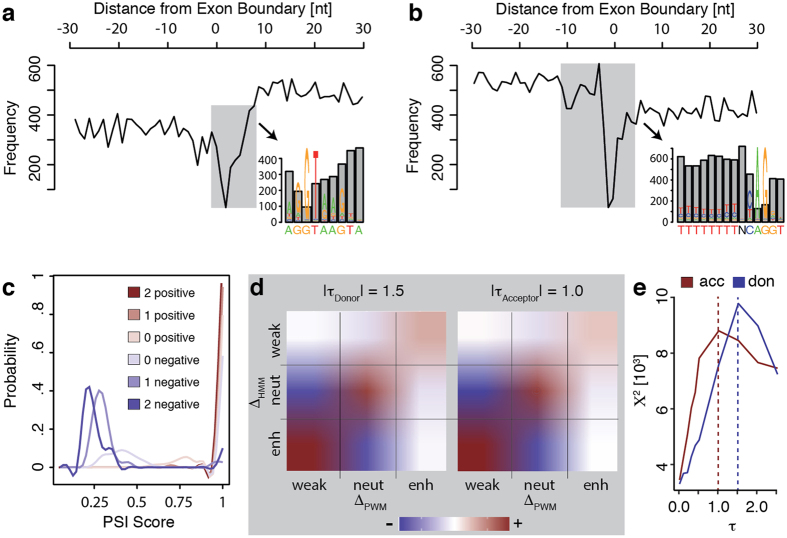
Genetic polymorphisms in splice sites. The distribution of nucleotide diversity (black curve) observed around splice donors (**a**) and splice acceptors (**b**) shows that polymorphisms are repressed in exons when compared to introns. Splice site dinucleotides are largely exempt of polymorphisms, and the frequency of polymorphisms observed in the remaining positions of the splice site motif scales about inversely with the information content of the consensus sequence (zoomed out areas). (**c**) Genetic variants with effects on the splicing score of alternative exons (0.2 < PSI < 0.8 in >75% of the population) are not randomly distributed. Variants for which the model predicts negative splicing effects target exons that are already mostly excluded in individuals that employ the reference splice site alleles (median PSI~ 0.4, light blue curve), whereas variants with positive effects occur in the splice sites of exons that are predominantly included in individuals with reference alleles (median PSI~ 0.75, light red curve). The predicted effect then gradually increases the observed PSI ex-/inclusion level in genotypes with one or both splice sites of an alternative exon accumulating negative/positive alleles (medium and dark blue/red curves). (**d**) The heatmaps show the agreement (from blue = depletion to red = enrichment) in the splice site score differences (Δ) caused by variants when comparing the scores computed by the HMM model employed herein (y-axis) with the scores obtained by a complementary PWM based model (x-axis), separately for splice donor (left panel) and acceptor sites (right panel). At the thresholds chosen to distinguish neutral from weakening/enhancing variants (|τ| = 1.5 for donor and |τ| = 1.0 for acceptor sites), the comparison between the classifications based on HMM predictions and those computed by PWMs yield very high enrichment scores (weakening = 72.87, neutral = 51.4, enhancing = 46.41 for donors, and weakening = 86.51, neutral = 76.18, enhancing = 33.17 for acceptors). (**e**) The Chi-Square Test statistic shows that indeed the best agreement between the PWM and the HMM scoring scheme is obtained at a threshold of |τ| = 1.5 (for donors, blue curve) respectively |τ| = 1.0 (for acceptors, red curve).

In order to investigate the molecular mechanisms that cause splicing variation between populations, we focused on variants that directly affect the affinity of annotated splice sites, considering an informative sequence of 9nt for splice donors including the GT dinucleotide, and 27nt for splice acceptors that include the AG dinucleotide and additionally the typical area of the preceding polypyrimidine tract (see Methods). When superimposing the 1000 Genomes DNA polymorphisms^32^ to the Gencode transcriptome version 12 reference transcriptome^38^, we find 10.7% (51,342 out of 477,880) of the annotated splice sites to harbor one (92% out of the 51,342) or multiple sequence polymorphisms in the core splice site motif (up to seven polymorph positions per splice site, Supplementary Fig. 1a). Splice sites exhibit a repression of indels (2.2% vs. 3.6% indels overall, p-value = 0.017). Also, allele frequencies of indels in splice sites are shifted to lower values (median frequency = 0.039 *vs*. 0.049 for indels not affecting splice sites, p-value = 0.11 Mann-Whitney-Wilcoxon (MWW) test) likely due to purifying selection against large genomic perturbations in functional elements^32^, albeit coding sequences with <0.5% indels exhibit even higher depletions. Furthermore, the frequency of single nucleotide polymorphisms (SNPs) occurring at certain positions of the splice site sequence is negatively correlated with the information content of the consensus motif, and the dinucleotides involved in the splicing reaction are mostly exempt of sequence polymorphisms (Fig. 1a,b).

Following earlier reports that genetic polymorphisms can directly affect splicing^39^,^40^, we computed splicing scores traditionally used in gene finding for evaluating the affinity of an RNA sequence to the splicing machinery in a systematic manner (Methods). Gene finders usually score potential splice sites in order to predict gene structures, however, we created a high-throughput tool for studying the effects of sequence variation in splice sites by employing these scoring schemes in an introspective manner, i.e. *a posteriori* given a set of splice sites. In technical terms our “Scorer” tool avoids the computation of a majority of hypothetical splice sites in a genome, and the associated overhead of filtering these predictions with respect to a given set of genes, and it additionally allows to provide a list of specific sequence variants based on the corresponding reference genome. For scoring splice sites, we employed the Hidden Markov Model (HMM) scoring matrices provided by the gene predictor GeneID^41^, which we further evaluate in the following with respect to their capabilities of introspectively evaluating splice site affinities based on the Geuvadis dataset.

Supplementary Fig. 1b shows that the implemented HMM model predicts different scores for donor and for acceptor sites, however, the scores computed for alternative splice sites and exons are lower than those for sites that are constitutively spliced, confirming earlier observations that modification of splicing can be driven by less efficient binding of splicing factors to the RNA sequence^42^. Turning to our Geuvadis phenotype data, we reassuringly observe several examples where the RNA-Seq splice-junction coverage supports our predictions of variant effects in the expected manner. In order to analyse how the predicted splice score of variants correlates with our RNA-seq data, we first studied the correlation between changes in the HMM score, measured as the difference between the score computed for the GRCh37 reference genome splice site sequence and the corresponding sequence with the annotated genomic variants, and percent-spliced-in (PSI) scores^43^ of alternatively included exons (0.2 < PSI < 0.8 in >80% of the individuals). We found that exons with variants that lower the computed splice site score (“negative” effects in Fig. 1c) exhibit low inclusion levels even in individuals carrying the reference allele (median PSI score 0.37), whereas variants with “positive” effects target preferentially the flanks of exons that are already relatively highly included employing the reference allele (median PSI score 0.76). The exon inclusion level then further gradually increases/decreases in individuals accumulating more variants with positive and respectively negative effects in their splice sites (Fig. 1c). In a nutshell, our analyses demonstrate that between individuals the usage of splice sites and of entire exons can be negatively as well as positively controlled by genetic variants.

To further evaluate and to classify the predicted HMM score changes, we compared them to corresponding predictions based on Position Weight Matrices (PWMs) from the complementary splice site discovery database SpliceRack^44^, providing the reference and variant splice site sequences collected by our Scorer tool. We analyzed different thresholds on the computed HMM score differences below which we do not consider a change in the score between the reference and the alternative allele of a splice site as biologically meaningful. We then classified sequence alterations for which we predict positive score changes above the chosen threshold as “enhancing” variants, and correspondingly negative score changes exceeding the threshold as “weakening” variants. Sequence polymorphisms that lead to score deviations less than the selected threshold are considered as “neutral” variants. When comparing for each threshold the classification by the HMM model to corresponding PWM-based calculations (Fig. 1d summarizes the systematic study shown in Supplementary Fig. 2), we observe clear enrichment of shared predictions in all three categories (i.e., “weakening”, “neutral”, and “enhancing” variants) at all thresholds, peaking at a threshold of 1.5 for donor and 1.0 for acceptor sites (p-value < 3e-323 at all thresholds, chi-squared test, Fig. 1e). The high agreement between both independent scoring schemes suggests that splice site scores are primarily a function of the analyzed sequence rather than the model employed to compute the score.

### Splice site disrupting variants are rare in the genome and in the gene pool

**Figure 2:**
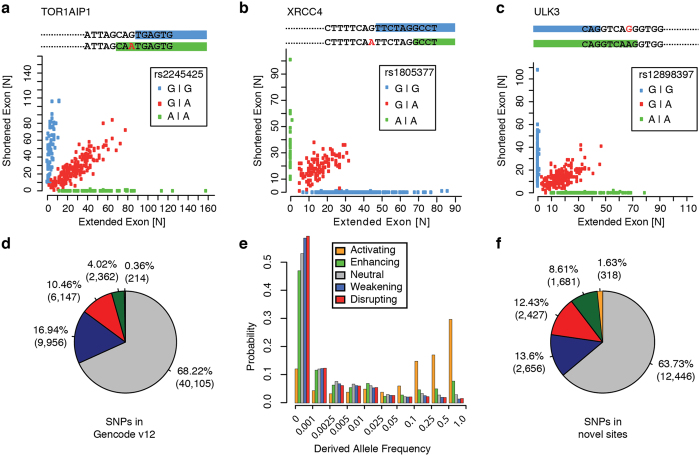
Distribution of different variant classes. (**a–c**) Scatter plots with examples for splice site switching triggered by splice site disrupting SNPs at the flanks of coding exons. The distribution of read counts at the extended (x-axis) and the shortened (y-axis) exon boundary is reported for all individuals carrying exclusively the reference allele (green), for individuals with homozygous SNP alleles (blue), and for heterozygous individuals (red). (**a**) A NAGNAG tandem acceptor site (delta = 3) in the TOR1AIP1 gene, (**b**) alternative acceptor sites (delta = 6) in the XRCC4 gene, and (**c**) alternative donor sites (delta = 6) in the URK3 gene. (**d**) The distribution of variants that stem from DNA polymorphisms in splice sites annotated by the Gencode v12 reference, classified accordingly by the differences in predicted splice site scores into disrupting (red), weakening (blue), neutral (gray), enhancing (green), and activating (orange) variants. Most sequence variants in splice sites are predicted to be neutral, and the Gencode reference splice sites harbor many more weakening and disrupting than enhancing and activating variants. (**e**) Derived allele frequencies (DAFs) of variants categorized according to the five different variant classes: alleles of enhancing variants (green bars) are deviating significantly (p-value ~ 2e-4, KS test), and alleles of activating variants (orange bars) even more significantly (p-value ~ 9e-5, KS test), from the distribution of allele frequencies of neutral variants (gray bars), enriching in higher abundant alleles. Weakening (blue bars) or disrupting variants (red bars) on the contrary accumulate more in low allele frequencies than neutral variants (p-value ~ 2e-3 and p-value ~ 2e-4, KS test). (**f**) An analogous pie chart as shown in (**d**), but for variants in novel splice sites of PNIs, exhibits relatively less neutral and weakening, but more enhancing, activating, and also disrupting variants.

In contrast to PWM estimates, the HMM model also pinpoints sequences with consecutive bases that have not been observed in the training set of splice sites used to establish the model (Methods). We therefore extend our classification to “activating” and “disrupting” variants for comparisons where the reference or alternative allele exhibit such splice site-absent sequences. Such variants include previously described SNPs that trigger alternative splice site usage between individuals by switching on/off cryptic splice sites. In these cases, homozygous individuals exhibit exclusively the use of one or the other exon boundary, whereas heterozygous individuals provide evidence of both splice sites being used (Fig. 2a–c).

Figure 2d summarizes the distribution of the different variant classes considered across all splice sites and individuals in the Geuvadis dataset and shows that the major part of SNPs in splice sites indeed fine-tunes the splicing activity, with a notably higher fraction of splicing weakening than enhancing variants (~17% *vs*. 4%). Disrupting variants (~10.5%) are less frequent, and actually only an exceptional minority (<0.5%) of SNPs in Gencode splice sites is activating. The differences in the relative proportion of disrupting *vs*. activating variants–and similarly also of weakening *vs*. enhancing variants–are presumptively provoked by a bias for functional alleles in the GRCh37 refs 45, 46. Since our classification of the variant effect depends by definition on the allele included in the human reference genome, the Geuvadis data suggests that in total ~22% of splice sites with genetic variants are modified in their splicing potential, about half of them severely by entirely disrupting the splicing activity, compared to a dominating subset of ~68% variants without predicted effects.

Our classification of genetic variants on splice sites is based on the effect of the non-reference allele, which corresponds to the derived allele when assuming that the reference genome represents the ancestral state. However, this is *a priori* not always the case. We therefore measured for variants in each variant class the global derived allele frequency (DAF), i.e., the frequency of the non-ancestral allele (Methods). Figure 2e shows that splicing-disrupting and also -weakening sequence polymorphisms are significantly more enriched (p-value ~ 2e-3 and 2e-4, Kolmogorov-Smirnov (KS) test) in low derived allele frequencies as compared to neutral variants. Enhancing variants on the contrary are shifted towards higher DAFs (p-value ~ 2e-4, KS test), and activating variants differ substantially in their global DAF distribution from all other splice site variant classes: 72% of activating SNPs exhibit DAFs >0.1 (p-value ~ 9e-5 compared to the distribution of neutral variants, KS test). Our results imply that activating variants are common variants for which the reference assembly of the human genome actually describes a low-frequency derived allele that disrupts the splice site.

To further estimate the degree to which the Geuvadis experiment can complement current knowledge about transcript annotation in LCLs, we superimposed split-mappings to the exon-intron structures of Gencode v12 to rescue putative novel introns (PNIs) that describe non-annotated exon-exon junctions (Methods). We found >64 million reads supporting ~2/3 of the annotated introns (222,862 out of 337,247 introns) and additionally ~14.7 million split-mappings that provide evidence for ~1.1 million PNIs. Although the overall size distribution of PNIs follows largely the one of introns annotated in the Gencode reference, a mixture of two lognormal distributions caused by distinct groups of short (~100nt) and long (~1,600nt) introns^47^, there are outliers of extremely short and long PNIs (Supplementary Fig. 3a). Most PNIs are predominantly observed in few individuals (Supplementary Fig. 3b) and also covered poorly by split-mappings in comparison to introns annotated in the Gencode reference (Supplementary Fig. 3c). However, PNIs also reflect many RNA-biology attributes similar to their annotated counterparts (Supplementary Fig. 4), the majority of PNIs (~74%) locate within annotated transcripts (i.e., “internal” events), and ~82% of them also employ at least one annotated splice site (Table 1a).

But also PNIs involving non-annotated (i.e., novel) splice sites and those that extend the transcript boundaries beyond the Gencode annotation (Table 1b) are supported well by complementary RNA-Seq data from the Encode project^48^, especially at higher thresholds of individual- and population-support (Table 2a). Like annotated splice sites (Fig. 1a), novel splice sites show evidence for genetic control of their splicing functionality, although at expectedly lower read support levels (Supplementary Fig. 3d). When clustering genetic variation caused by 19,528 variants in novel GT/AG splice sites from PNIs confirmed by >150 individuals according to the effects on splicing, we find amongst the variant groups a ranking similar to the one of splice sites annotated in the Gencode reference, but with highly significant shifts towards fewer neutral (p-value ~ e-30, Fisher Exact test) and weakening (p-value ~ e-29), but more enhancing (p-value ~ e-125), activating variants (p-value ~ e-65, Fig. 2f). In the context of our previous observations on the bias of the human reference genome in favor of more functional elements, these differences can be explained by non-annotated PNIs showing a reduced bias for functional reference alleles (Fig. 2d *vs*. 2f). However, we also observe an increase in the relative proportion of disrupting variants (p-value~ e-14), which could reflect that disrupted splice junctions are underrepresented in the Gencode annotation by their generally lower expression levels^26^.

### RNA editing as a splice site modulator

**Figure 3:**
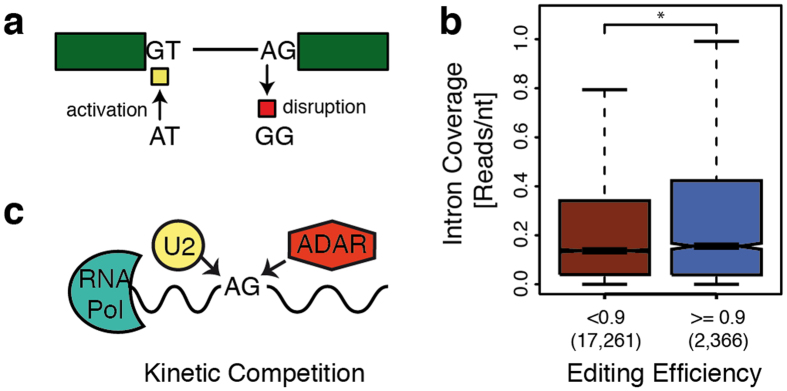
ADAR catalyzed RNA editing predicted in splice sites. (**a**) A-to-I RNA editing catalyzed by the ADAR complex can disrupt (red marker) the canonical U2 splice acceptor dinucleotide AG, as for instance predicted in the MDM2 gene. In contrast, the GT splice donor dinucleotide can be created from AT, which is usually recognized as a donor only by the minor spliceosome, as predicted in the RASGRP3 gene. (**b**) Incomplete RNA editing (red boxplot) of splicing disrupting bases is associated with significantly lower intron coverage by RNA-Seq reads (y-axis) than observed for disrupted sites that are exhaustively edited (blue boxplot, p-value = 0.03, MWW test). The observed intron read coverage can serve as a proxy for the number of introns retained when the splicing machinery fails to recognize the correspondingly edited, thus disrupted, splice site. (**c**) The cartoon sketches a competitive model for the cotranscriptional processes of splicing and RNA-editing, where components of the splicing machinery (e.g., U2) compete with the ADAR enzyme complex for the splice site substrate.

Next, we employed our methodology to analyze Gencode splice sites for the impact of potential RNA editing events catalyzed by the ADAR enzyme complex (Methods), which produces A-to-I conversions that are represented by A-to-G transitions in the RNA-Seq data^49^. Reassuringly, our approach calls substantially fewer splice sites with putative RNA editing polymorphisms than with genetic polymorphisms (<0.01% *vs*. 10.7%). Only two of the 39 editing events we predict to incur in the region of annotated splice sites are contained in the complementary RADAR-2 database^50^, however, this database includes data from studies that intentionally select against editing events in annotated splice sites^51^,^52^,^53^. In contrast to genetic variants (Fig. 2d), more than twice the proportion of edited nucleotides (~68% *vs*. 32%) disrupt their harboring splice site, which can be expected by mechanistic restrictions when considering the possible sequence alterations of ADAR editing in the canonical dinucleotides of annotated sites (Fig. 3a). Consequently, we observe 28 A-to-G transitions that disrupt the AG acceptor dinucleotide, whereas the only activation event we predict for ADAR editing incurs by conversion of a donor AT dinucleotide, usually employed in a very limited set of introns spliced by the minor spliceosome^54^.

Our data in Supplementary Fig. 5a further suggests that RNA editing targets significantly shorter introns (median 607nt vs. 1,881nt in constitutive introns), and particularly RNA editing events that disrupt splicing activities are limited to very short introns (median 522.5nt vs. 972nt in the other introns with edited sites, p-value ~1.1e-09, MWW test). Supplementary Table 1 also summarizes that, according to the Gencode reference transcriptome, most of the splice sites (28 of 41 sites) that are affected by RNA editing are alternatively spliced, which interestingly leads predominantly to retaining the entire intron (in 18 of 28 introns with edited sites). Indeed, we also observe in the Geuvadis dataset substantial amounts of reads from introns flanked by sites with predicted editing events (Supplementary Fig. 5b), in agreement with recent reports concluding that the ADAR complex can sterically block the splicing machinery from accessing the RNA substrate^55^.

Unlike the binary state of variants encoded by the genome, RNA editing constitutes a more gradual trait that has been reported to vary across individuals, transcript sequences and gene expression levels^56^. Interestingly, we also find in the Geuvadis data that the editing efficiency in splicing disrupting events anti-correlates with the splicing efficiency, as introns flanked by disrupted sites that are exhaustively edited (>0.9 of non-reference bases) exhibit higher intron read coverages and therefore more retained introns (Fig. 3b). We do not observe this difference for non-disruptive editing events (Supplementary Fig. 5c). These results support complementary observations of splicing^57^ and also RNA editing^58^ being co-transcriptionally competing processes (Fig. 3c). Our findings suggest that both molecular processes are often temporally coordinated, as also reported by complementary evidence^55^,^59^, and that RNA editing can guide splice site choice in particular genes and species^60^,^61^,^62^,^63^.

### Genetic diversity in polyadenylation signals

Beyond splicing, we also investigated the impact of inter-individual DNA variability on polyadenylation. To obtain 3′ end information we predicted 52,349 putative cleavage sites (PCSs) from read mappings that align partly with the genomic sequence and exhibit poly-A tails (Methods). The number of PCSs found with higher read support levels decreases rapidly (Supplementary Fig. 6a), but independently of the expression rate of the underlying transcript (Supplementary Fig. 6b). In our further analyses we focus on the conservative subset of 21,102 PCSs supported by ≥2 reads, which are still twice as many as identified in previous studies^28^,^64^. These PCSs exhibit a high degree of overlap with annotated 3′ UTRs (71.4%), especially within a distance of 50 nt from 3′ transcript ends annotated in Gencode (66%), and they are highly supported by complementary RNA-Seq data from the Encode Project (Table 1b).

**Figure 4:**
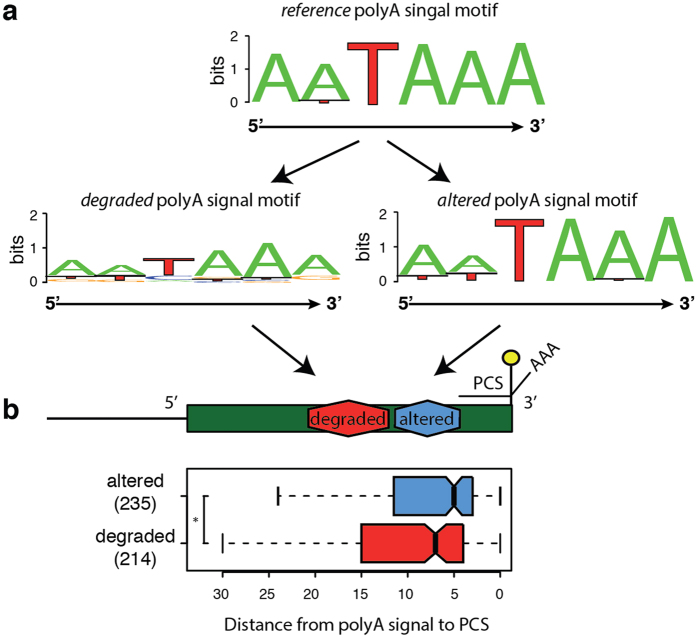
Genetic variants in poly-A motifs. (**a**) The sequence logo of poly-A motifs in the human reference genome sequence GRCh37 reproduces well the distribution of nucleotides from earlier reports (sequence logo at the top). Genetic variants can change the poly-A motif to another sequence that is known to act as a poly-A signal (i.e. “altered” motifs, sequence logo at the bottom-left of the panel), or they can disrupt the poly-A motif such that the variant sequence no longer corresponds to any reported poly-A signal (“degraded” motifs, sequence logo to the bottom-right). (**b**) When analysing the distribution of distances between poly-A signals and the closest PCS, the 235 poly-A motifs altered by genetic variants (blue distribution) localize slightly but significantly (p-value 0.016 MWW test) closer to the PCS than the 214 poly-A motifs that are degraded by SNPs (red distribution).

Scanning the genomic sequence around these PCSs (Methods), we identified for 96.3% of them sequences that agree with earlier described poly-A motifs, and the nucleotide distribution of their consensus also matches earlier reports^36^. For those that coincide with polyA-signals provided by the Gencode annotation, we additionally analyzed the degree up to which genetic variation affects the composition of the poly-A motif. Most poly-A motifs are exempt of SNPs, but Fig. 4a shows 235 events of SNPs that are reproducing known poly-A signals and therefore overall maintain the consensus profile (“altered motifs”, left panel in Fig. 4a) in contrast to 214 polymorphisms that produce sequences unknown to function as poly-A signals that distort the consensus and therefore likely disrupt the affinity of the site to the CPSF (“degraded” motifs, right panel in Fig. 4a). Interestingly, we observe that poly-A motifs that are degraded by genetic variation locate marginally but significantly further away from the PCSs (Fig. 4b), indicating a different relevance of the CPSF for 3′-end formation. Summing up, we collected the Geuvadis RNA-Seq evidence for splice sites, introns and cleavage sites that are not annotated in the Gencode v12 reference, and we exhaustively characterized the implications of genetic variation also in these novel elements.

## Discussion

In this study we employed the genetic diversity annotated for 462 individuals from the 1000 Genomes project, to compose a genome-wide catalogue of genetic polymorphisms in annotated splice sites and to estimate their potential effects on splicing based on the sequence changes in splice site motifs. In this light we consider the landscape of inter-individual variants described by the large-scale Geuvadis experiment as a natural source of mutagenesis experiments from which we deduce rules for the regulation of splicing. Due to their important functional role, splice sites are generally depleted for genetic polymorphisms, and our results suggest an even higher level of selective constraints in the splice site dinucleotides than in the adjacent exon sequences. Employing HMM scoring models established in gene finding, we implemented a tool that allows to score the splicing potential of splice sites and their variants. We evaluate the computed score by an alternative scoring model based on PWMs, and we compare the results produced by either method to establish a rationale to classify the changes observed in splicing scores in five classes (i.e., disrupting, weakening, neutral, enhancing, and activating variants). From a computational point of view, we contribute to forthcoming studies along the same lines by making our programs to compute splicing scores for reference and variant sites publicly available.

Based on these score predictions, the mechanistic impact of genetic variation on splice sites is often of subtle nature, for instance modulating the inclusion level of alternative exons, but can also be rather severe. We describe variants that activate or disrupt entirely the splicing activity, providing examples from the Geuvadis Project where SNPs switch intron splicing allele-specifically on or off. Although RNA-editing can also affect splicing, we find that ADAR-edited splice sites are comparatively rare, however, with a higher degree of disrupting variants caused by A-to-G substitutions in the canonical AG dinucleotide of the acceptor site. Our analyses suggest that RNA-editing targets mainly short introns of evolutionary rather old genes, most of the edited sites are already known to be alternatively used and many are related to intron retention. The Geuvadis dataset shows a substantial amount of intronic reads in introns with edited sites, as expected in the proposed model under which the ADAR complex makes the RNA molecule inaccessible to the splicing machinery, and in concordance with the computed splice site scores the RNA-Seq coverage is even higher in introns with splice sites that are predicted to disrupt splicing activity. We also find that the RNA-Seq read coverage of introns with splice sites disrupted by RNA-editing increases when editing levels rise close to the complete substitution of the genomic base, whereas this is not observed in introns with edited sites that are still predicted to be functional. Altogether, the computational models we apply to combined DNA- and RNA-sequencing at a population scale support multiple aspects of RNA editing postulated by previous observations in limited gene sets.

Allele frequencies from the 1000 Genomes project show that most of the genetic variation affecting splicing stems from rare alleles in the population, but we discover also a small set of common polymorphisms that actually describe a functional splice site in contrast to a splicing-defective reference sequence, which shows that relying exclusively on the reference genome in gene annotation and polymorphism effect estimation may be problematic in specific cases. In fact, the combined sequencing depth of hundreds of samples and billions of reads provides us with the power to detect thousands of transcribed elements that are not annotated in the Gencode reference annotation, including novel introns (PNIs) and cleavage sites (PCSs). The majority of these previously undetected elements are also discovered in complementary RNA-Seq data from the Encode project and exhibit attributes similar to the biology of their annotated counterparts. Many of them occur only in few individuals, which may be the reason why they are absent from existing annotations, but they may still be important determinants of personal transcriptomes by contributing to the genetic makeup of each individual.

Employing these novel elements predicted from the phenotype data, we show that PNIs exhibit a higher proportion of activating as well as disrupting variants, indicating that the absence of their splicing can be tolerated more often. These conclusions are in agreement with our observations of comparatively low splicing and population frequencies for PNIs. We also find that genetic polymorphisms potentially disrupt poly-A signals, especially in cases where the CPSF recognition site localizes slightly further away from the PCS. In a nutshell, our results are certainly limited because RNA-Seq in the Geuvadis experiment have been obtained from a single cell type per individual, namely lymphoblastoid cell lines, and we expect that our observations will be extended in the future with more population-scale tissue data becoming available. However, our study demonstrates a hitherto less explored potential for mechanistic studies on the inter-individual variability and population diversity in RNA-processing that can be derived by combined RNA- and DNA-sequencing.

## Methods

Supplementary Fig. 7 shows an overview of all resources employed and the analyses carried out for this work, employing the analyses detailed in the following.

### Computing splicing scores

Following traditional approaches in gene finding^41^, we employ computational splice site models that comprise an informative sequence of 9nt for splice donors (interval [−2; 7]), and 27nt for splice acceptors—from −24 to +3 including additionally the typical area of the upstream polypyrimidine tract^65^. We first apply these models to the splice sites annotated in the GENCODE version 12 reference transcriptome, and subsequently also to novel introns (PNIs, see below) as well as predicted RNA-editing in splice sites (see below). To estimate splicing efficiency of polymorphisms, the splice site sequence composition is represented by a second order Markov Model^66^,^67^. Under this model, sequences with a higher degree of similarity to the consensus bind more tightly to the corresponding factors of the splicing machinery^68^,^69^, and therefore are more frequently observed as authentic splice sites^70^,^71^. We then compute the log-odds “splicing score” and compare the scores of sequences derived from splice site variants with the score of the corresponding splice site reference sequence in the human genome assembly GRCh37. Our scoring algorithm is implemented in the Scorer tool of the Astalavista framework available at http://scorer.sammeth.net, which we employed using the command: astalavista -t scorer -i gencode_v12.gtf -c GRCh37_sequences_folder –gid geneid.human.070123.param –vcf population_variants.vcf -f population_variant_scores.vcl

where geneid.human.070123.param is the GeneID parameters file for the human genome, downloaded from ftp://genome.crg.es/pub/software/geneid/human.070123.param.

### Comparison of HMM scores with PWM scores

Hidden Markov Model (HMM) scores were calculated with our Astalavista Scorer tool as described above. Position Weight Matrix (PWM) scores were calculated by running the FIMO^72^ motif scanning tool with default parameters on the splice site DNA sequences retrieved with the Astalavista Scorer tool, using PWMs from the SpliceRack database^44^. The motif score assigned by FIMO was used as the PWM score. For both approaches, score differences Δ_HMM_ and Δ_PWM_ were calculated by subtracting the reference sequence (RS) score from the variant sequence (VS) score, with negative score differences suggesting splice site “weakening” variants while positive differences imply splice site “enhancing” variants. As the PWM scores exhibited a trimodal distribution separated by minima at ~+/−6, we classified all score differences between −6 and +6 as “neutral” variants (Supplementary Fig. S2a). We subsequently varied the “neutral” threshold for the HMM score differences between 0 and +/−2.5, and we determined the degree of classification agreement as enrichment between the two scoring schemes using the chi-square test from the R statistical program^73^. The enrichment is measured as the standardized residuals of the chi-square test, i.e., an enrichment of x means that the observed frequency of coincidences is x times the standard deviation away from the expected frequency of coincidences between both models.

### Classification of sequence variants in splice sites

SNPs that increase/decrease the splicing score of a reference splice site sequence above/below the previously determined threshold (|τ| = 1.5 for donors, and |τ| = 1.0 for acceptors) are classified as “enhancing”/“weakening” variants. In the cases where either the GRCh37 genome or the splice site variant reproduces a sequence that is absent from the training set of our model, we assume that the sequence does not represent a functional splice site and consider the corresponding variants as “activating”/“disrupting” the splice site potential. All other sequence variations that do not change the splicing score more than |τ| are “neutral” polymorphisms. We employed the global derived allele frequencies (DAFs) computed for the non-reference alleles by the 1000 Genomes Project.

### Prediction of RNA editing in splice sites

We employed the samtools (version 0.1.18) mpileup tool in combination with the bundled vcfutils.pl script^74^ to call sequence polymorphisms from RNA-Seq reads by the following command: samtools mpileup -C0 -m3 -F0.0002 -E -d999999 -q20 -DSuf hg19.fa -b inputBams | bcftools view -cgv - | vcfutils.pl varFilter -Q25 -d3 -D4999500 -a2 -w10 -W10 -10.0001 -21e-400 -30 -40.0001 -p > variants.vcf

This pipeline produces from the Geuvadis RNA-Seq mappings (“inputBams”) a list of variants (“variants.vcf”), employing the mpileup standard parameters for disabling the adjustment of mapQ (-C0) and for the minimum fraction of gapped reads (-F0.002), but allowing a higher per-BAM depth (-d999999), to attribute for the unequal read coverage in genes with different expression levels, and requiring a higher mapQ (-q20) for mappings to be considered during calling. The corresponding parameters (-D4999500 and -Q25) were also adjusted in the vcfutils.pl filtering script, where we additionally increased the stringency for polymorphisms to not locate up to 10nt next to a gapped position (-w10 and -W10). Subsequently, we merged the calls from 421 individuals with non-imputed genotypes in the Phase2 dataset of the 1000 Genomes Project^32^, removing polymorphisms with a median coverage of <10 at called sites, with <10 samples showing the called non-reference base, and with a variant quality of <100 assigned by SAMtools. We thus obtained 8,479 predictions polymorphisms, of which 7,770 (91.6%) correspond to 1000 Genomes genotype variants employed by the Geuvadis Project:

http://www.ebi.ac.uk/arrayexpress/experiments/E-GEUV-1/files/genotypes/

Considering the transcription directionality of each respective gene, 39 of the remaining 709 non-genomic polymorphisms correspond to A-to-G variants that modify in total 41 introns annotated by the Gencode reference (Supplementary Table 1).

### Prediction of putative novel introns (PNIs)

We rescue PNIs from split-mapped RNA-Seq reads that indicate non-annotated alternative 5′ or 3′ splice sites within proximity of up to 30 nt to an annotated exon boundary, considering only properly paired mappings with a mapping quality of at least 150, an edit distance ≤6, and an insert-size of ≤1,000,000 nt. We then superimpose PNIs to the exon-intron structures of the Gencode v12 annotation, and we employ our earlier described definition to classify the patterns of alternative splicing events implied by these novel introns^75^.

### Prediction of putative cleavage sites (PCSs)

To identify putative cleavage sites, we employ unmapped reads containing a poly-A tail (or a poly-T head) that pinpoint the cleavage site in poly-adenylated mRNAs. After trimming the reads for these subsequences, filtering them by a minimum informative length (>25nt after trimming) and removing low complexity reads (i.e., read sequences with an [A] and [T] content ≥80%), we obtain ~24 million reads of which 685,351 map uniquely to the genome and indicate 52,349 putative cleavage sites (PCSs). This can be summarized by the following commands, using the trimest tool^76^: samtools view -f 4 $BAMFILE | awk ‘{if($10 !~ /\./&& (($10~/AAAA$/) || ($10 ~/^TTTT/))){cnt++;print “>“cnt”\n”$10}}’ | trimest -filter -minlength=5 -fiveprime Y -mismatches=1 | perl FastaToTbl.pl | awk –f selByLenAndContent.awk | perl TblToFasta.pl>$OUTFILE

selByLenAndContent.awk: {len=length($2);cntA=cntT=0; for(i=0;i<len+1;i++){if(substr($2,i,1)==“A”) {cntA++;} if(substr($2,i,1)==“T”) {cntT++;}}rA=cntA/len;rT=cntT/len;rr=rA+rT;if((rr < 0.8) && length($2)>25){print;}}

This pipeline receives as input a BAM file (BAMFILE) and produces a file with polyA reads already trimmed and selected. The scripts FastaToTbl and TblToFasta convert from tabular format to Fasta format. We consider a PCS predicted from the Geuvadis RNA-Seq data to be confirmed if we can extract a corresponding PCS from the Encode dataset that intersects in the genomic region to which the non poly-adenylated parts of supporting reads align. This analysis can be summarized by the following command using BedTools^77^: windowBed -a gencode.polyA.sites.bed -b./geuvadis.polyA.bed -w 50 -c | awk ‘{if($7>0)print}’

### Finding poly-A signals

In order to identify poly-A motifs for previously identified PCSs, we use a recursive approach similar to an earlier proposed method^37^. We employ 13 hexamer motifs that have been identified as potential binding sites of the CPSF^36^,^37^, i.e. AATAAA, ATTAAA, TATAAA, AGTAAA, AAGAAA, AATATA, AATACA, CATAAA, GATAAA, AATGAA, TTTAAA, ACTAAA, AATAGA. This list of hexamers is ranked by the frequency with which each motif is observed, with AATAAA being the most and AATAGA the least frequent poly-A motif in the human transcriptome. We then scan the DNA sequences of 50 nt around the previously predicted PCSs in a top-down approach, starting with searching for the most frequently occurring hexamer; if a corresponding hexamer sequence is found, we record its position, otherwise we continue scanning with next most frequent motif until all of the 13 known poly-A motifs have been tested.

## Additional Information

**How to cite this article**: Ferreira, P. G. *et al*. Sequence variation between 462 human individuals fine-tunes functional sites of RNA processing. *Sci. Rep.* **6**, 32406; doi: 10.1038/srep32406 (2016).

## Supplementary Material

Supplementary Information

## Figures and Tables

**Table 1:**
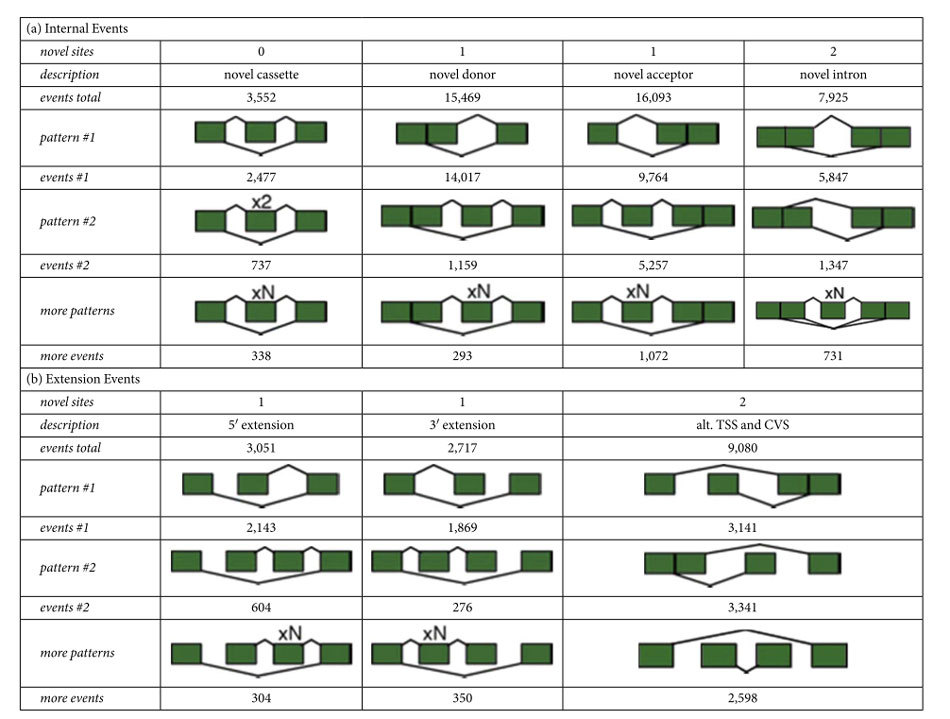
Alternative splicing implied by putative novel introns (PNIs).

The table summarises novel alternative splicing events implied by superimposing the 21,761 Gencode PNIs supported by >150 individuals to the transcript structures of the Gencode reference annotation. The events have been grouped according to their localisation within the transcript body (i.e., “internal events”, Table 1a) or beyond the transcript extremities (“extension events”, Table 1b). The 1^st^ row presents the number of novel splice sites, i.e. the splice sites of the PNI that are not annotated in the Gencode reference, described in each category (i.e., the column). The 2^nd^ row provides the total count of such events. Rows 3–8 show the two most frequently observed event patterns (“pattern #1” and “pattern #2”) in the category and a summary of remaining patterns (“more patterns”), with the corresponding number of single events observed for each pattern. (a) Most internal PNIs link novel splice sites to an existing one (~73%), less frequently introns employ two novel sites (~18%), and novel combinations of existing sites are rather exceptional (~8%). (b) In contrast, PNIs employing novel splice sites upstream of the annotated transcription start site (TSS) or downstream of the annotated cleavage site (CVS) are more frequently combinations of two novel splice sites (~62% *vs*. ~59%).

**Table 2:** Mutual confirmation of novel transcriptional elements in Geuvadis and Encode RNA-Seq data.

	Geuvadis	Encode	Novel
(a) PNIs			
All	1,068,786	62,5%	400,697
>0 in all populations	205,649	94,9%	10,553
>150 individuals	21,761	97,8%	469
>300 individuals	6,660	97,9%	139
>450 individuals	846	97,4%	22
(b) PCSs			
all PCSs ≥2 mappings	21,102	62,8%	7,856
not overlapping 3′ UTR	6,032	40,2%	3,607
overlapping 3′ UTR	15,070	86,6%	2,017

The table presents the number of different (subsets of) novel transcriptional elements (rows) predicted from the Geuvadis experiments (column 2), the proportion of these elements that is additionally confirmed by Encode RNA-Seq reads (column 3), and the number of non-overlapping (i.e., novel) elements in Geuvadis as compared to Encode. (a) Nearly 2/3 (~63%) of the putative novel introns (PNIs) in Geuvadis are also contained in the 34,926,167 Encode PNIs. Applying more restrictive population support thresholds on the PNIs leads to high confirmation rates (>97%). (b) Putative cleavage sites (PCSs) with a support of ≥2 RNA-Seq reads show similar a base level (~63%) of overlap between the Geuvadis data set and the 160,331 PCSs correspondingly rescued from the Encode data. For PCSs outside annotated 3′ UTRs the confirmation rate decreases (~40%), whereas PCSs in 3′ UTR regions are strongly supported by Encode data (~87%).

## References

[b1] Black D. L. Mechanisms of alternative pre-messenger RNA splicing. Annu. Rev. Biochem. 72, 291–336 (2003). 12626338 10.1146/annurev.biochem.72.121801.161720

[b2] Black D. L., Chabot B. & Steitz J. A. U2 as well as U1 small nuclear ribonucleoproteins are involved in premessenger RNA splicing. Cell 42, 737–750 (1985). 2996775 10.1016/0092-8674(85)90270-3

[b3] Wahle E. & Kühn U. The mechanism of 3′ cleavage and polyadenylation of eukaryotic pre-mRNA. Prog. Nucleic Acid Res. Mol. Biol. 57, 41–71 (1997). 9175430 10.1016/s0079-6603(08)60277-9

[b4] Colgan D. F. & Manley J. L. Mechanism and regulation of mRNA polyadenylation. Genes Dev. 11, 2755–2766 (1997). 9353246 10.1101/gad.11.21.2755

[b5] Curado J., Iannone C., Tilgner H., Valcárcel J. & Guigó R. Promoter-like epigenetic signatures in exons displaying cell type-specific splicing. Genome Biol. 16, 236 (2015). 26498677 10.1186/s13059-015-0797-8 PMC4619081

[b6] Derrien T., Guigó R. & Johnson R. The Long Non-Coding RNAs: A New (P)layer in the ‘Dark Matter’. Front. Genet. 2, 107 (2011). 22303401 10.3389/fgene.2011.00107 PMC3266617

[b7] Wilusz J. E., Sunwoo H. & Spector D. L. Long noncoding RNAs: functional surprises from the RNA world. Genes Dev. 23, 1494–1504 (2009). 19571179 10.1101/gad.1800909 PMC3152381

[b8] Tilgner H. . Nucleosome positioning as a determinant of exon recognition. Nat. Struct. Mol. Biol. 16, 996–1001 (2009). 19684599 10.1038/nsmb.1658

[b9] Papasaikas P., Tejedor J. R., Vigevani L. & Valcárcel J. Functional splicing network reveals extensive regulatory potential of the core spliceosomal machinery. Mol. Cell 57, 7–22 (2015). 25482510 10.1016/j.molcel.2014.10.030

[b10] Krawczak M., Reiss J. & Cooper D. N. The mutational spectrum of single base-pair substitutions in mRNA splice junctions of human genes: causes and consequences. Hum. Genet. 90, 41–54 (1992). 1427786 10.1007/BF00210743

[b11] Alipanahi B., Delong A., Weirauch M. T. & Frey B. J. Predicting the sequence specificities of DNA- and RNA-binding proteins by deep learning. Nat. Biotechnol. 33, 831–838 (2015). 26213851 10.1038/nbt.3300

[b12] Xiong H. Y. . The human splicing code reveals new insights into the genetic determinants of disease. Science 347, 1254806 (2015). 25525159 10.1126/science.1254806 PMC4362528

[b13] Garcia-Blanco M. A., Baraniak A. P. & Lasda E. L. Alternative splicing in disease and therapy. Nat. Biotechnol. 22, 535–546 (2004). 15122293 10.1038/nbt964

[b14] Faustino N. A. & Cooper T. A. Pre-mRNA splicing and human disease. Genes Dev. 17, 419–437 (2003). 12600935 10.1101/gad.1048803

[b15] Singh R. K. & Cooper T. A. Pre-mRNA splicing in disease and therapeutics. Trends Mol. Med. 18, 472–482 (2012). 22819011 10.1016/j.molmed.2012.06.006 PMC3411911

[b16] Acedo A. . Comprehensive splicing functional analysis of DNA variants of the BRCA2 gene by hybrid minigenes. Breast Cancer Res. 14, R87 (2012). 22632462 10.1186/bcr3202 PMC3446350

[b17] Rahman M. A. . HnRNP L and hnRNP LL antagonistically modulate PTB-mediated splicing suppression of CHRNA1 pre-mRNA. Sci. Rep. 3, 2931 (2013). 24121633 10.1038/srep02931 PMC3796306

[b18] Vibe-Pedersen K., Kornblihtt A. R. & Baralle F. E. Expression of a human alpha-globin/fibronectin gene hybrid generates two mRNAs by alternative splicing. EMBO J. 3, 2511–2516 (1984). 6096127 10.1002/j.1460-2075.1984.tb02165.x PMC557721

[b19] Kwan T. . Heritability of alternative splicing in the human genome. Genome Res. 17, 1210–1218 (2007). 17671095 10.1101/gr.6281007 PMC1933514

[b20] Zhang X., Zou F. & Wang W. Efficient Algorithms for Genome-wide Association Study. ACM Trans. Knowl. Discov. Data 3, 19:1–19:28 (2009).

[b21] Fraser H. B. & Xie X. Common polymorphic transcript variation in human disease. Genome Res. 19, 567–575 (2009). 19189928 10.1101/gr.083477.108

[b22] Kwan T. . Tissue effect on genetic control of transcript isoform variation. PLoS Genet. 5, e1000608 (2009). 19680542 10.1371/journal.pgen.1000608 PMC2719916

[b23] Lu Z.-X., Jiang P. & Xing Y. Genetic variation of pre-mRNA alternative splicing in human populations. Wiley Interdiscip. Rev. RNA 3, 581–592 (2012). 22095823 10.1002/wrna.120 PMC3339278

[b24] Monlong J., Calvo M., Ferreira P. G. & Guigó R. Identification of genetic variants associated with alternative splicing using sQTLseekeR. Nat. Commun. 5, 4698 (2014). 25140736 10.1038/ncomms5698 PMC4143934

[b25] Ongen H. & Dermitzakis E. T. Alternative Splicing QTLs in European and African Populations. Am. J. Hum. Genet. 97, 567–575 (2015). 26430802 10.1016/j.ajhg.2015.09.004 PMC4596912

[b26] Rivas M. A. . Human genomics. Effect of predicted protein-truncating genetic variants on the human transcriptome. Science 348, 666–669 (2015). 25954003 10.1126/science.1261877 PMC4537935

[b27] Montgomery S. B. . Transcriptome genetics using second generation sequencing in a Caucasian population. Nature 464, 773–777 (2010). 20220756 10.1038/nature08903 PMC3836232

[b28] Pickrell J. K. . Understanding mechanisms underlying human gene expression variation with RNA sequencing. Nature 464, 768–772 (2010). 20220758 10.1038/nature08872 PMC3089435

[b29] Stranger B. E. . Population genomics of human gene expression. Nat. Genet. 39, 1217–1224 (2007). 17873874 10.1038/ng2142 PMC2683249

[b30] Cheung V. G. . Mapping determinants of human gene expression by regional and genome-wide association. Nature 437, 1365–1369 (2005). 16251966 10.1038/nature04244 PMC3005311

[b31] Dimas A. S. . Common regulatory variation impacts gene expression in a cell type-dependent manner. Science 325, 1246–1250 (2009). 19644074 10.1126/science.1174148 PMC2867218

[b32] Abecasis G. R. . An integrated map of genetic variation from 1,092 human genomes. Nature 135, 0–9 (2012). 10.1038/nature11632 PMC3498066 23128226

[b33] Lappalainen T. . Transcriptome and genome sequencing uncovers functional variation in humans. Nature 501, 506–511 (2013). 24037378 10.1038/nature12531 PMC3918453

[b34] ’t Hoen P. A. C. . Reproducibility of high-throughput mRNA and small RNA sequencing across laboratories. Nat. Biotechnol. 31, 1015–1022 (2013). 24037425 10.1038/nbt.2702

[b35] Zhang X. H.-F., Leslie C. S. & Chasin L. a. Dichotomous splicing signals in exon flanks. Genome Res. 15, 768–779 (2005). 15930489 10.1101/gr.3217705 PMC1142467

[b36] Beaudoing E., Freier S., Wyatt J. R., Claverie J. M. & Gautheret D. Patterns of variant polyadenylation signal usage in human genes. Genome Res. 10, 1001–1010 (2000). 10899149 10.1101/gr.10.7.1001 PMC310884

[b37] Tian B., Hu J., Zhang H. & Lutz C. S. A large-scale analysis of mRNA polyadenylation of human and mouse genes. Nucleic Acids Res. 33, 201–212 (2005). 15647503 10.1093/nar/gki158 PMC546146

[b38] Harrow J. . GENCODE: the reference human genome annotation for The ENCODE Project. Genome Res. 22, 1760–1774 (2012). 22955987 10.1101/gr.135350.111 PMC3431492

[b39] Graveley B. R. The haplo-spliceo-transcriptome: common variations in alternative splicing in the human population. Trends Genet. 24, 5–7 (2008). 18054116 10.1016/j.tig.2007.10.004 PMC2372159

[b40] Zhang W. . Identification of common genetic variants that account for transcript isoform variation between human populations. Hum. Genet. 125, 81–93 (2009). 19052777 10.1007/s00439-008-0601-x PMC2665168

[b41] Guigó R., Knudsen S., Drake N. & Smith T. Prediction of gene structure. J. Mol. Biol. 226, 141–157 (1992). 1619647 10.1016/0022-2836(92)90130-c

[b42] Ast G. How did alternative splicing evolve? Nat. Rev. Genet. 5, 773–782 (2004). 15510168 10.1038/nrg1451

[b43] Wang E. T. . Alternative isoform regulation in human tissue transcriptomes. Nature 456, 470–476 (2008). 18978772 10.1038/nature07509 PMC2593745

[b44] Sheth N. . Comprehensive splice-site analysis using comparative genomics. Nucleic Acids Res. 34, 3955–3967 (2006). 16914448 10.1093/nar/gkl556 PMC1557818

[b45] Lander E. S. . Initial sequencing and analysis of the human genome. Nature 409, 860–921 (2001). 11237011 10.1038/35057062

[b46] Olivier M. . A high-resolution radiation hybrid map of the human genome draft sequence. Science 291, 1298–1302 (2001). 11181994 10.1126/science.1057437

[b47] Lim L. P. & Burge C. B. A computational analysis of sequence features involved in recognition of short introns. Proc. Natl. Acad. Sci. USA 98, 11193–11198 (2001). 11572975 10.1073/pnas.201407298 PMC58706

[b48] Djebali S. . Landscape of transcription in human cells. Nature 489, 101–108 (2012). 22955620 10.1038/nature11233 PMC3684276

[b49] Nishikura K. Functions and regulation of RNA editing by ADAR deaminases. Annu. Rev. Biochem. 79, 321–349 (2010). 20192758 10.1146/annurev-biochem-060208-105251 PMC2953425

[b50] Ramaswami G. & Li J. B. RADAR: a rigorously annotated database of A-to-I RNA editing. Nucleic Acids Res. 42, D109–D113 (2014). 24163250 10.1093/nar/gkt996 PMC3965033

[b51] Kleinman C. L., Adoue V. & Majewski J. RNA editing of protein sequences: a rare event in human transcriptomes. RNA 18, 1586–1596 (2012). 22832026 10.1261/rna.033233.112 PMC3425774

[b52] Ramaswami G. . Identifying RNA editing sites using RNA sequencing data alone. Nat. Methods 10, 128–132 (2013). 23291724 10.1038/nmeth.2330 PMC3676881

[b53] Ramaswami G. . Accurate identification of human Alu and non-Alu RNA editing sites. Nat. Methods 9, 579–581 (2012). 22484847 10.1038/nmeth.1982 PMC3662811

[b54] Wu Q. & Krainer A. R. AT-AC pre-mRNA splicing mechanisms and conservation of minor introns in voltage-gated ion channel genes. Mol. Cell. Biol. 19, 3225–3236 (1999). 10207048 10.1128/mcb.19.5.3225 PMC84117

[b55] Licht K., Kapoor U., Mayrhofer E. & Jantsch M. F. Adenosine to Inosine editing frequency controlled by splicing efficiency. Nucleic Acids Res. 10.1093/nar/gkw325 (2016). PMC5291252 27112566

[b56] Fumagalli D. . Principles Governing A-to-I RNA Editing in the Breast Cancer Transcriptome. Cell Rep. 13, 277–289 (2015). 26440892 10.1016/j.celrep.2015.09.032 PMC5326813

[b57] Tilgner H. . Deep sequencing of subcellular RNA fractions shows splicing to be predominantly co-transcriptional in the human genome but inefficient for lncRNAs. Genome Res. 22, 1616–1625 (2012). 22955974 10.1101/gr.134445.111 PMC3431479

[b58] Rodriguez J., Menet J. S. & Rosbash M. Nascent-seq indicates widespread cotranscriptional RNA editing in Drosophila. Mol. Cell 47, 27–37 (2012). 22658416 10.1016/j.molcel.2012.05.002 PMC3409466

[b59] Laurencikiene J., Källman A. M., Fong N., Bentley D. L. & Ohman M. RNA editing and alternative splicing: the importance of co-transcriptional coordination. EMBO Rep. 7, 303–307 (2006). 16440002 10.1038/sj.embor.7400621 PMC1456888

[b60] Rueter S. M., Dawson T. R. & Emeson R. B. Regulation of alternative splicing by RNA editing. Nature 399, 75–80 (1999). 10331393 10.1038/19992

[b61] Jin Y. . RNA editing and alternative splicing of the insect nAChR subunit alpha6 transcript: evolutionary conservation, divergence and regulation. BMC Evol. Biol. 7, 98 (2007). 17597521 10.1186/1471-2148-7-98 PMC1919356

[b62] Jones A. K. . Splice-variant-and stage-specific RNA editing of the Drosophila GABA receptor modulates agonist potency. J. Neurosci. 29, 4287–4292 (2009). 19339622 10.1523/JNEUROSCI.5251-08.2009 PMC6665385

[b63] Grohmann M. . Alternative splicing and extensive RNA editing of human TPH2 transcripts. PLoS One 5, e8956 (2010). 20126463 10.1371/journal.pone.0008956 PMC2813293

[b64] Fu Y. . Differential genome-wide profiling of tandem 3′ UTRs among human breast cancer and normal cells by high-throughput sequencing. Genome Res. 21, 741–747 (2011). 21474764 10.1101/gr.115295.110 PMC3083091

[b65] Coolidge C. J., Seely R. J. & Patton J. G. Functional analysis of the polypyrimidine tract in pre-mRNA splicing. Nucleic Acids Res. 25, 888–896 (1997). 9016643 10.1093/nar/25.4.888 PMC146492

[b66] Blanco E., Parra G. & Guigó R. Using geneid to identify genes. Curr. Protoc. Bioinformatics Chapter 4, Unit 4.3 (2007). 10.1002/0471250953.bi0403s18 18428791

[b67] Hull J. . Identification of common genetic variation that modulates alternative splicing. PLoS Genet. 3, e99 (2007). 17571926 10.1371/journal.pgen.0030099 PMC1904363

[b68] Nelson K. K. & Green M. R. Mechanism for cryptic splice site activation during pre-mRNA splicing. Proc. Natl. Acad. Sci. USA 87, 6253–6257 (1990). 2143583 10.1073/pnas.87.16.6253 PMC54511

[b69] Zamore P. D., Patton J. G. & Green M. R. Cloning and domain structure of the mammalian splicing factor U2AF. Nature 355, 609–614 (1992). 1538748 10.1038/355609a0

[b70] Ohshima Y. & Gotoh Y. Signals for the selection of a splice site in pre-mRNA. Computer analysis of splice junction sequences and like sequences. J. Mol. Biol. 195, 247–259 (1987). 3656413 10.1016/0022-2836(87)90647-4

[b71] Brunak S., Engelbrecht J. & Knudsen S. Prediction of human mRNA donor and acceptor sites from the DNA sequence. J. Mol. Biol. 220, 49–65 (1991). 2067018 10.1016/0022-2836(91)90380-o

[b72] Grant C. E., Bailey T. L. & Noble W. S. FIMO: scanning for occurrences of a given motif. Bioinformatics 27, 1017–1018 (2011). 21330290 10.1093/bioinformatics/btr064 PMC3065696

[b73] Team, R. C. R: A language and environment for statistical computing. R Foundation for Statistical Computing, Vienna, Austria. 2013 (2014).

[b74] Li H. . The Sequence Alignment/Map format and SAMtools. Bioinformatics 25, 2078–2079 (2009). 19505943 10.1093/bioinformatics/btp352 PMC2723002

[b75] Sammeth M., Foissac S. & Guigó R. A General Definition and Nomenclature for Alternative Splicing Events. PLoS Comput. Biol. 4, e1000147 (2008). 18688268 10.1371/journal.pcbi.1000147 PMC2467475

[b76] Rice P., Longden I. & Bleasby A. EMBOSS: the European Molecular Biology Open Software Suite. Trends Genet. 16, 276–277 (2000). 10827456 10.1016/s0168-9525(00)02024-2

[b77] Quinlan A. R. & Hall I. M. BEDTools: a flexible suite of utilities for comparing genomic features. Bioinformatics 26, 841–842 (2010). 20110278 10.1093/bioinformatics/btq033 PMC2832824

