# Supplementary material for: Sequence variation between 462 human individuals fine-tunes functional sites of RNA processing
Source: Sci Rep. 2016 Sep 12;6:32406. doi: 10.1038/srep32406 (PMC5019111; doi:10.1038/srep32406)
Supplement: Supplementary Information [file srep32406-s1.pdf]

Sequence variation between 462 human individuals fine-tunes functional sites of RNA processing

Pedro G. Ferreira, Martin Oti, Matthias Barann, Thomas Wieland, Suzana Ezquina, Marc R Friedländer, Manuel A Rivas, Anna Esteve-Codina, The Geuvadis Consortium, Philip Rosenstiel, Tim M Strom, Tuuli Lappalainen, Roderic Guigó, Michael Sammeth

Supplementary Information

- Supplementary Information
  - Supplementary Figures
    - Figure S1: Splice site variants and splicing scores
    - Figure S2: Evaluation of splice site scores
    - Figure S3: Characteristics of putative novel introns (PNIs)
    - Figure S4: Novel exon boundaries implied by putative novel introns (PNIs)
    - Figure S5: Attributes of introns and splice sites with RNA editing calls
    - Figure S6: Characteristics of putative cleavage sites (PCSs)
    - Figure S7: Layout of the study
  - Supplementary Tables
    - Table S1: RNA edited splice sites

Supplementary Figures

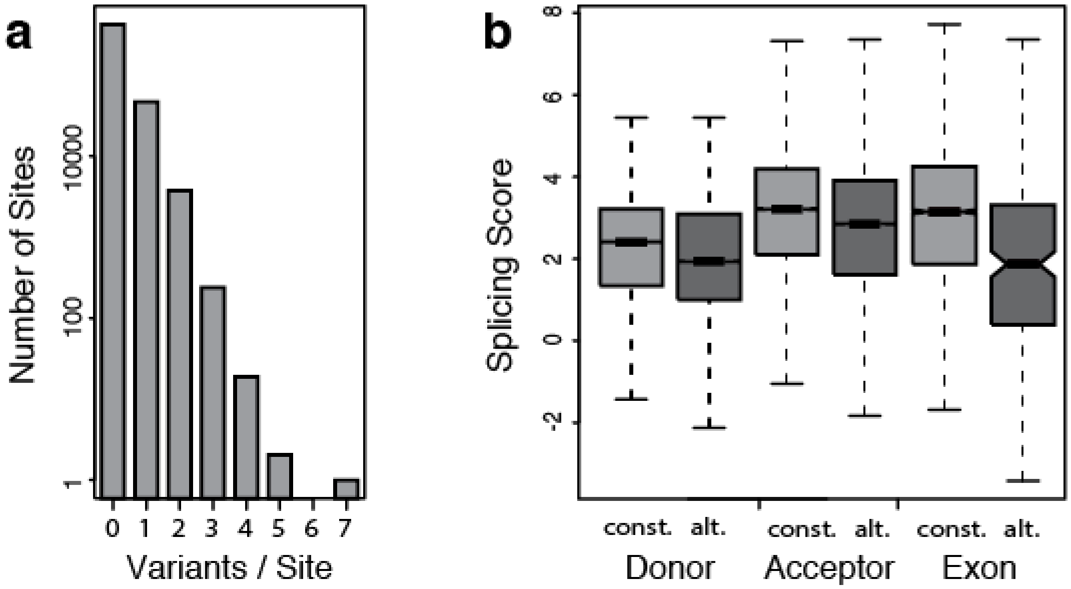

Figure S1: Splice site variants and splicing scores

(a) The number of splice sites that harbour multiple DNA polymorphisms decreases exponentially. (b) The distributions of HMM log-odd scores (splicing scores) computed for splice donor sites (green), splice acceptor sites (red) and exons (blue) confirm that constitutively spliced elements (light gray) exhibit generally higher scores their than alternatively spliced counterparts (dark gray). Splicing scores for exons have been computed by summing the splicing score calculated for each of their splice sites independently. Constitutive splice sites have been distinguished from alternative ones by the transcript annotations in the Gencode v12 reference, and exons with a PSI (percentage-spliced-in) value of >0.9 in >90% of the Geuvadis individuals have been considered constitutively spliced, otherwise they were classified as alternatively spliced exons.

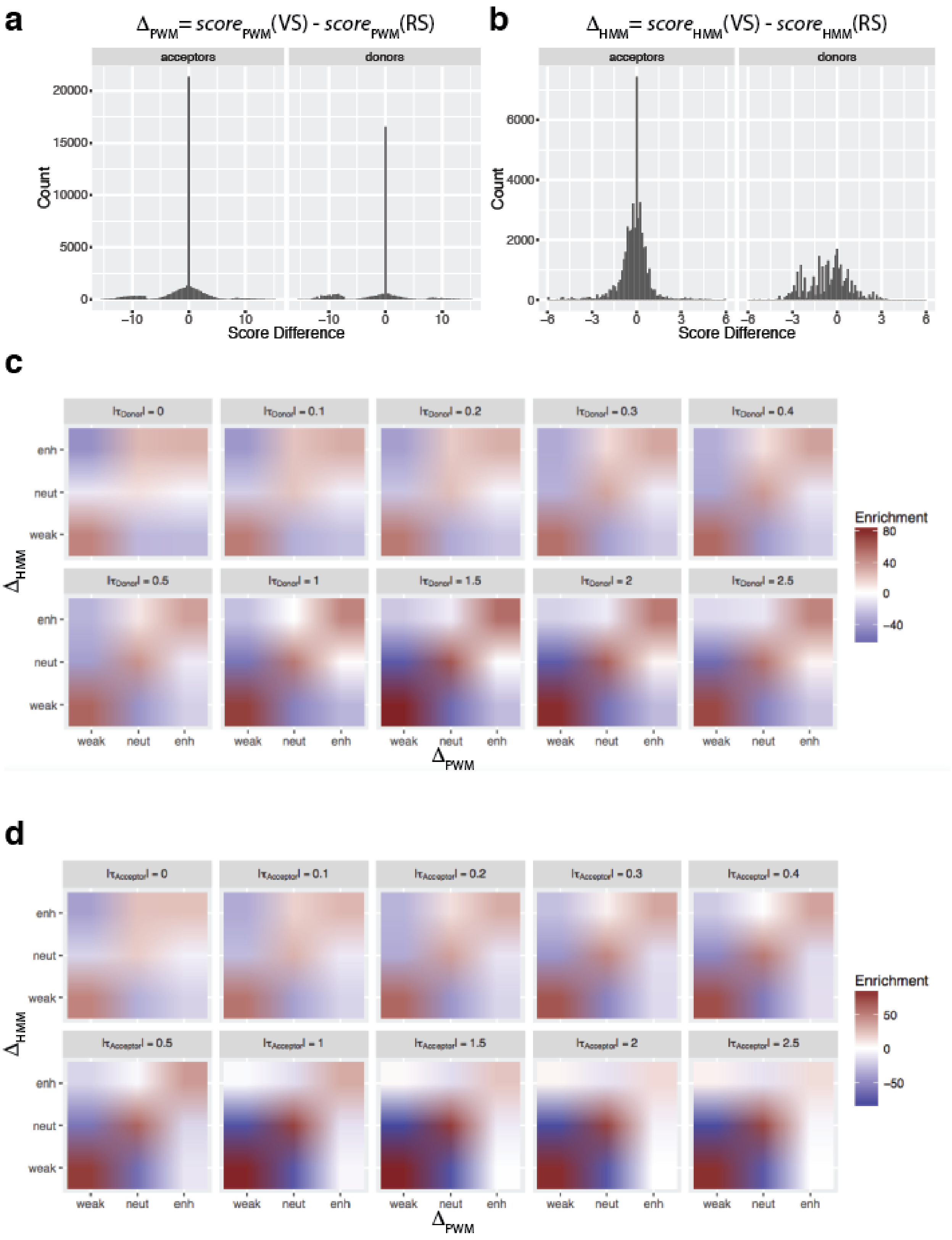
a
Δ_PWM = score_PWM(VS) - score_PWM(RS)
acceptors
donors
Count
20000
15000
10000
5000
0
−10
0
10
Score Difference
b
Δ_HMM = score_HMM(VS) - score_HMM(RS)
acceptors
donors
Count
6000
4000
2000
0
−6
−3
0
3
6
Score Difference
c
|τ_Donor| = 0
|τ_Donor| = 0.1
|τ_Donor| = 0.2
|τ_Donor| = 0.3
|τ_Donor| = 0.4
|τ_Donor| = 0.5
|τ_Donor| = 1
|τ_Donor| = 1.5
|τ_Donor| = 2
|τ_Donor| = 2.5
Δ_HMM
enh
neut
weak
Δ_PWM
Enrichment
80
40
0
−40
d
|τ_Acceptor| = 0
|τ_Acceptor| = 0.1
|τ_Acceptor| = 0.2
|τ_Acceptor| = 0.3
|τ_Acceptor| = 0.4
|τ_Acceptor| = 0.5
|τ_Acceptor| = 1
|τ_Acceptor| = 1.5
|τ_Acceptor| = 2
|τ_Acceptor| = 2.5
Δ_HMM
enh
neut
weak
Δ_PWM
Enrichment
50
0
−50

Figure S2: Evaluation of splice site scores

(a) The histogram shows the score differences Δ_{PWM} obtained when subtracting the score for the reference splice site (RS) from the score of the variant splice site (VS), both computed based on PWMs. The distribution of Δ-values shows three distinct classes, separating enhancing variants ($\Delta_{PWM} > 6$) and weakening variants ($\Delta_{PWM} < -6$) from neutral variants ($-6 \leq \Delta_{PWM} \leq 6$). (b) In contrast, the histogram of the analogously computed Δ_{HMM} score differences shows a more continuous distribution. Heatmap plots reveal the enrichment measured as standardized residuals of the chi-square test for (c) donor sites, and (d) acceptor sites when comparing PWM score categories (x-axis) to systematically evaluated categories based on HMM scores (y-axis). Variant effects have been classified according to different thresholds $|\tau| = \{0.0, ..., 2.5\}$ (shown at the top of each panel) on the Δ_{HMM} values: all $\Delta_{HMM} < -|\tau|$ are classified as weakening variants, all $\Delta_{HMM} > |\tau|$ are classified as enhancing variants, and all remaining Δ_{HMM} score changes are considered as neutral variants. Subsequently, the agreement between both scoring schemes was assessed by the enrichment of predictions from either method in the classification categories, scaling from negative (blue) to positive (red) scores. The systematic benchmark shows high enrichment in classifications that are mutually confirmed by PWM and HMM Δ-values (i.e., in the weakening, in the neutral, and in the enhancing categories along the diagonal), especially at higher thresholds (i.e., $|\tau| \geq 1.5$ for donors, and $|\tau| \geq 1.0$ for acceptors).

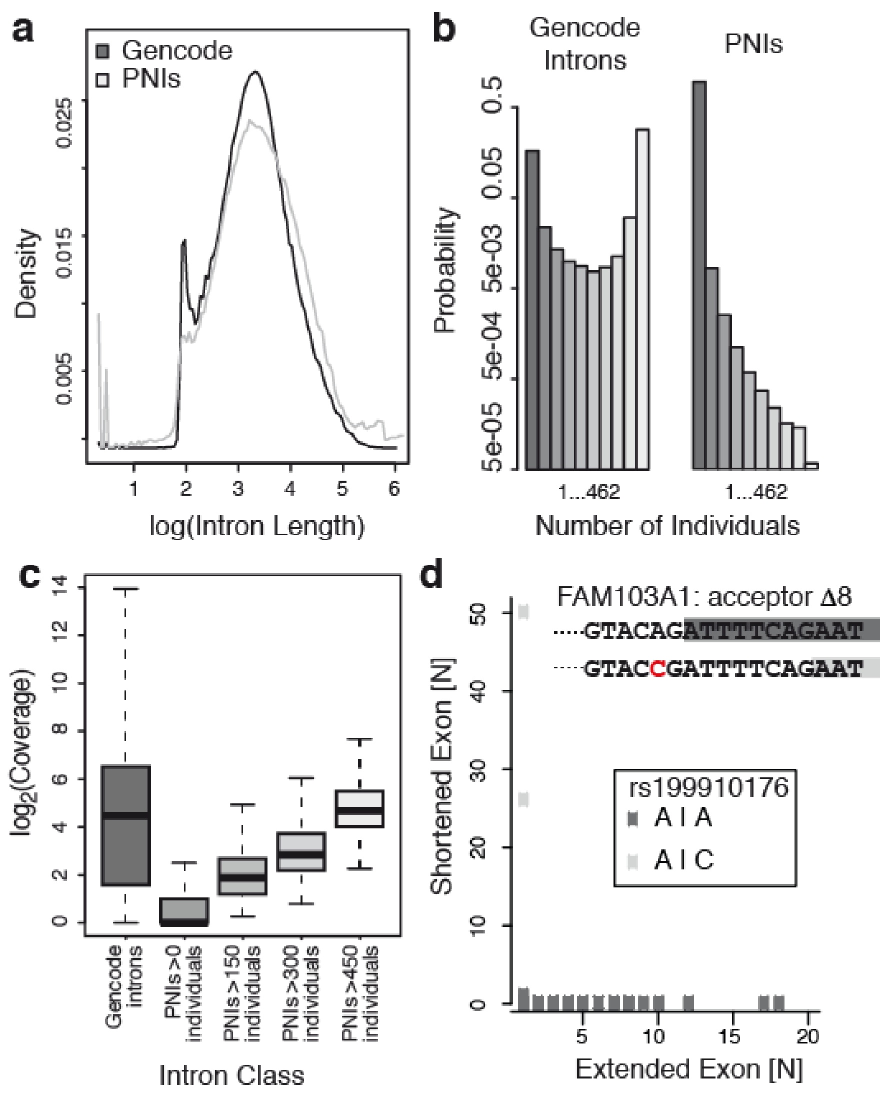

Figure S3: Characteristics of putative novel introns (PNIs)

(a) The length distribution of PNIs (grey curve) follows largely the histogram of intron sizes annotated in the Gencode reference transcriptome (black curve), with two main peaks for short introns (~100nt) and long introns (~1,600nt). PNIs exhibit additional outliers created by unusually short or long introns. (b) Introns annotated in the Gencode reference show a U-shaped distribution of population support, with many introns being supported by few or most individuals of the Geuvadis dataset. Whereas, most of the PNIs are minority events, observed only in a small proportion of individuals. (c) The split-mapped read coverage of PNIs correlates well with the population support, i.e., the number of individuals supporting the PNI. (d) The example shows a genetic polymorphism that creates alternative splicing at a novel cryptic splice site, by an A>C SNP disrupting the annotated upstream acceptor (chr15: 83,657,760) and enabling splicing at a latent downstream acceptor site 8nt downstream, which is not annotated in the Gencode reference (chr15: 83,657,768).

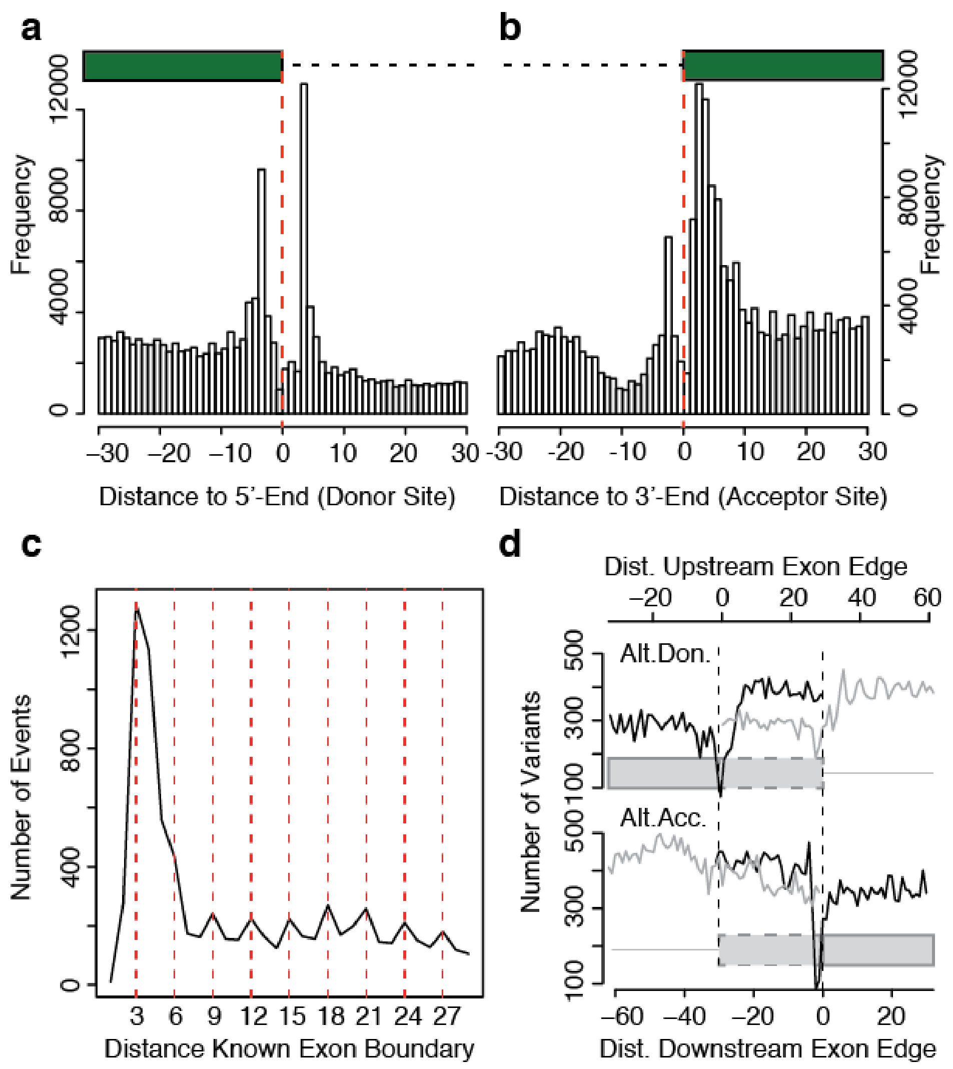

Figure S4: Novel exon boundaries implied by putative novel introns (PNIs)

Panel (a) and (b) show preferred locations of novel exon boundaries, as determined by the number of RNA-Seq mappings (y-axis), relative to the position of annotated exon boundaries (x-axis). These preferences confirm expectations based on characteristics intrinsic to the splice site motifs, at +4 for donor and at (–3) for acceptor sites. We also observe alternative splice site creation repressed in a region of about (-10) nt before the acceptor dinucleotide, which corresponds to the distance of a typical branch point, a region known to be depleted of adenine bases that could be confounded with the true branch site. (c) The distance of novel exon boundaries shows a periodicity of three (dashed red lines), implying influences by the coding sequence. (d) Novel exon boundaries that extend annotated exons (dashed box) also exhibit changes in the frequency of DNA polymorphisms (grey curve), similar to the one observed at annotated exon flanks (black curve).

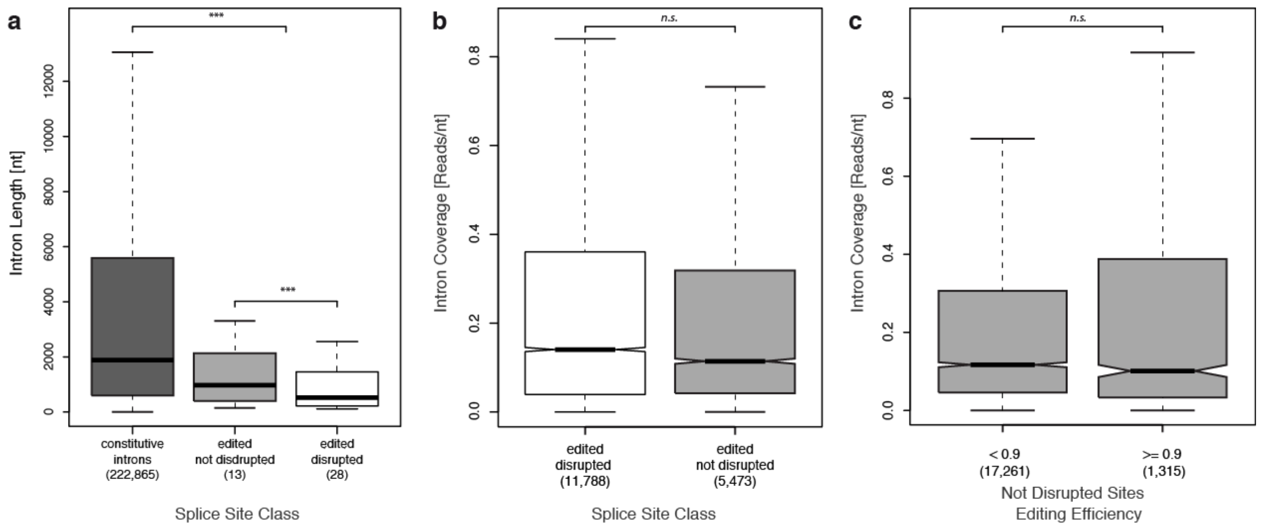

Figure S5: Attributes of introns and splice sites with RNA editing calls

(a) Constitutive introns with a median length of 1,881nt (dark gray box, mean= 7,246nt) are significantly longer than introns with predicted editing events in their splice site (constituted by the light gray and white distribution: median= 607nt, mean= 1,056nt, p-value ~8.8e-06, MWW test). Also introns with edited but not disrupted sites (median= 972nt, mean= 1,554nt) are still longer than introns with splice sites that are predicted to be disrupted by RNA editing (median= 522.5nt, mean= 842.9nt; p-value ~1.1e-09, MWW test). The number in parentheses below each class of introns shows the number of introns analyzed. (b) The intron coverage (y-axis) is only marginally higher in introns with splice sites that are presumptively disrupted by RNA editing than in introns with splice sites that are affected but not disrupted by editing events (median 0.14 vs. 0.11 reads/nt, p-value ~0.058, MWW test). (c) Also the read coverage levels of introns that are lowly and highly edited do not differ significantly from each other (median 0.12 vs. 0.10 reads/nt, p-value ~0.052, MWW test).

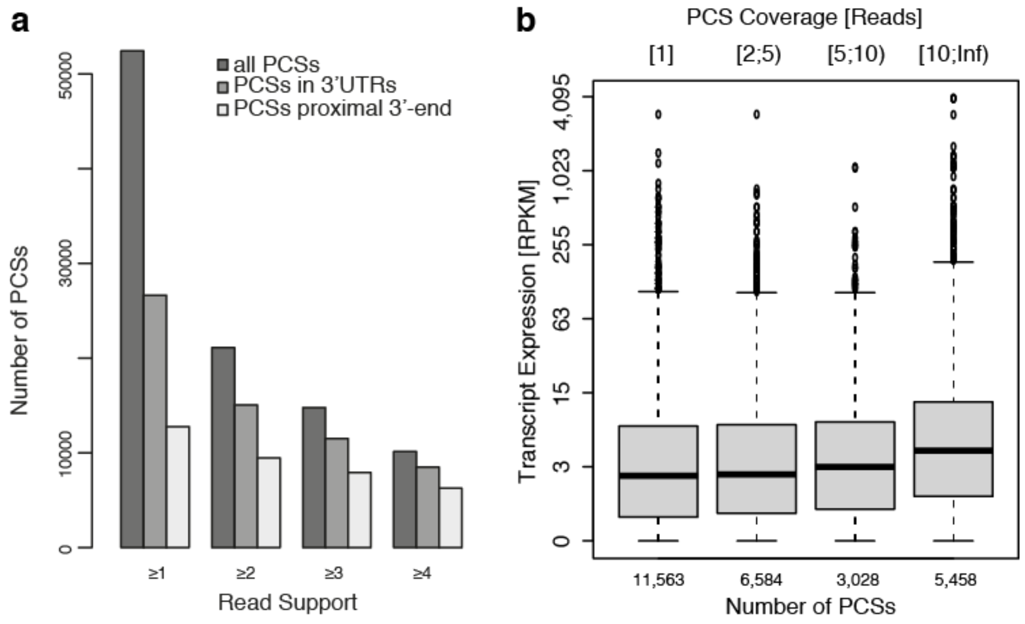

Figure S6: Characteristics of putative cleavage sites (PCSs)

(a) PCSs that are confirmed exclusively by one read are often predicted outside of annotated 3' UTRs (dark grey bar). In contrast, more conservative PCS predictions with >1 supporting read (x-categories) locate relatively more often in 3' UTRs (medium grey bars) or within 50nt proximity of annotated cleavage sites (light grey bar). (b) Expression levels (i.e., distributions of RPKM values, y-axis) in the genes overlapping PCSs are rather constant when considering PCSs with different read support levels (x-categories), showing that the PCS coverage is determined by polyadenylation efficiency rather than by expression.

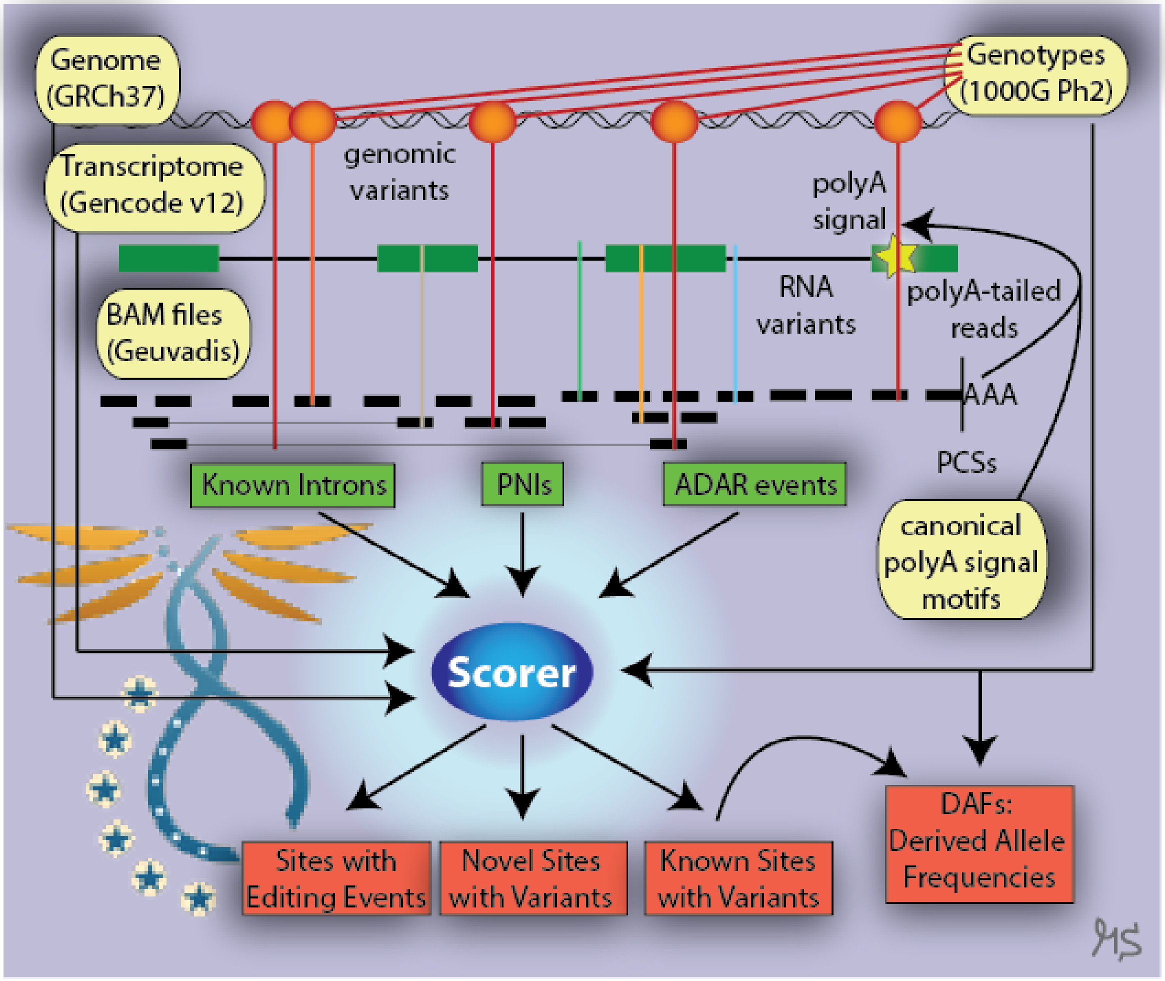

Figure S7: Layout of the study

Yellow boxes: For our study, we employed the human genome assembly GRCh37, the Gencode v12 transcriptome annotation, 13 hexamer sequences described in literature as the most abundant polyA signals in human, genotypes annotated by Phase 2 of the 1000 Genomes Project (http://www.ebi.ac.uk/arrayexpress/experiments/E-GEUV-1/files/genotypes/) and the Geuvadis RNA-Seq reads from quality controlled individuals and their corresponding mapping locations in the genome (http://www.ebi.ac.uk/arrayexpress/files/E-GEUV-1/processed/). *Green boxes*: From these read mappings, we collected all expressed introns known in the Gencode annotation and we rescued putative novel introns (PNIs) as well as putative cleavage sites (PCSs), following the corresponding protocols described in our Methods section. Furthermore, we called putative RNA editing events from mapped reads by polymorphisms that are not of genomic origin and that describe A-to-G transitions as caused by the ADAR pathway. The effects of sequence variation in polyadenylation signals has been analyzed by evaluating the altered polyA signals according to the reference polyA signal sequences. *Red boxes*: Variants in splice sites were evaluated by the Scorer tool we developed, which we used to predict the effect

of DNA and RNA polymorphisms in known as well as in novel splice sites. Employing allele frequencies determined by the 1000 Genomes Project for each variant, we additionally estimated derived allele frequencies (DAFs) for the variants of different categories.

Supplementary Tables

Table S1: RNA edited splice sites

The table characterizes 41 different splice sites influenced by ADAR editing events. The predicted effect (column "Fx") of each RNA modification (columns "Chr" and "Pos") is categorized according to the splice score into disrupting (DR), weakening (WK), neutral (NT), enhancing (EH) and activating (AV) RNA editing events. The "Site" column shows the modified splice site sequence, with the putatively edited base highlighted by a capital "G" letter, the splice site dinucleotide marked in red, and the adjacent exon boundary represented by the symbol "|" in the sequence. The column "AS" (i.e., alternative splicing) shows whether the Gencode reference annotates no alternative splicing at all ("NA"), intron retention ("IR"), and respectively alternative donor or alternative acceptor sites ("AD" / "AA") in the representative intron. In the case of the GSTK1 gene, the retained intron maintains the annotated reading frame (marked by an asterisk). Note that most of the genes with predicted RNA editing events are essential and related to ribosomal functions, RNA processing or splicing, and located in intracellular organelles.

Gene	***Transcript ID***	***Chr***	***Pos***	***Site***	***Fx***	***Intron start***	***Intron end***	***AS***
ENO1	OTTHUMT00000004945.1	1	8,930,571	aatttgatttgattgttcct tcc**Gg**|gtgtc	DR	8,930,569	8,931,950	AA
RPL11	OTTHUMT00000008168.1	1	24,021,288	tgtg|gtatga**G**ta	NT	24,021,281	24,022,288	AD
MCL1	OTTHUMT00000084402.1	1	150,549,969	tgtgcttttcttttttgttt tct**Gg**|gatgg	DR	150,549,967	150,550,720	NA
RASGRP3	OTTHUMT00000325341.1	2	33,741,712	gcat|**G**tatctttt	AV	33,741,711	33,745,019	AD
SP140L	OTTHUMT00000374538.1	2	231,264,957	aga**G**|gtaagtgac	EH	231,264,957	231,265,670	IR
QARS	OTTHUMT00000345693.1	3	49,140,844	cagctccttgtgcctatgct tct**Gg**|gagag	DR	49,140,842	49,141,064	IR

TKT	OTTHUMT00000 350356.1	3	53,265,568	ctgtcctcccgtgttgggtc tgcGg|gggta	DR	53,265,566	53,267,172	IR
RFS23	OTTHUMT00000 370358.1	5	81,573,673	aagaactaaaatatttaatt tttGg|gcaag	DR	81,573,671	81,574,139	AA
ERAP1	ENST00000 414384.2	5	96,110,490	gaatttttatGcttatttgt tgtag|tgaat	NT	96,110,475	96,111,938	IR
CD74	OTTHUMT00000 374177.1	5	149,785,886	aaccttctgttacttcctcc cacGg|cccat	DR	149,785,884	149,786,444	NA
CD74	OTTHUMT00000 374177.1	5	149,786,525	cttaccctcgttcctgtccc cacGg|ctccc	DR	149,786,523	149,786,715	IR
MAT2B	OTTHUMT00000 374193.1	5	162,943,522	attatttgcttttattcttc tctGg|gagct	DR	162,941,000	162,943,524	NA
DDX39B	OTTHUMT00000 259083.1	6	31,498,705	agtgctaccctctgtctccc tccGg|gtggc	DR	31,498,703	31,498,830	AA
TAP1	OTTHUMT00000 076087.2	6	32,814,841	acag|gtgGggcag	WK	32,813,562	32,814,845	NA
SFT2D1	OTTHUMT00000 043062.2	6	166,738,085	attgctctgtttttgctgtt gttGg|tggca	DR	166,738,083	166,739,620	AA
GSTK1	OTTHUMT00000 327143.1	7	142,962,352	tcctgctgtcttctcttctt cccGg|aatga	DR	142,962,185	142,962,354	IR*
STOML2	OTTHUMT00000 052273.1	9	35,101,559	gtggggcttacacctctttt tccGg|gaacg	DR	35,101,557	35,101,707	NA
TXN	OTTHUMT00000 053614.1	9	113,013,097	tcag|gtGtgtagc	WK	113,007,123	113,013,100	AA
P4HA1	OTTHUMT00000 048602.1	10	74,774,049	gaatttactgtgtttttatt cacGg|aaaga	DR	74,774,047	74,776,604	NA
FUT11	OTTHUMT00000 048692.1	10	75,538,589	gcttaccctgtttgaccttt tgcag|aaaGg	EH	75,535,916	75,538,586	AA
RPS13	OTTHUMT00000 387320.2	11	17,099,026	cgagactgcttctctccccG ggaag|ggcct	EH	17,099,024	17,099,166	IR
RPS13	OTTHUMT00000 387321.1	11	17,099,026	acctcgagactgcttctctc cccGg|gaagg	DR	17,099,020	17,099,166	IR
PTPN6	OTTHUMT00000 400019.1	12	7,066,815	tccccacccgaccctccctt tccGg|aacaa	DR	7,065,731	7,066,817	NA
PFDN5	OTTHUMT00000 405372.1	12	53,690,026	gtgtttgtcttcattgcttt cacGg|ggaaa	DR	53,689,725	53,690,028	IR
PCBP2	OTTHUMT00000 407073.1	12	53,862,559	ttgcttgctctcttctgtct tttGg|caggt	DR	53,861,627	53,862,561	IR
PCBP2	OTTHUMT00000 407545.1	12	53,862,559	cttgctctcttctgtctttt Ggcag|gtttg	EH	53,861,627	53,862,564	IR
ATP5B	OTTHUMT00000 408380.1	12	57,038,952	ttgg|gtGagtaga	NT	57,038,739	57,038,955	NA

MDM2	OTTHUMT00000 402676.1	12	69,237,519	tgtctgtctcaataaatggc caa**Gg**|ggatt	DR	69,237,044	69,237,521	IR
PABPN1	OTTHUMT00000 071766.4	14	23,792,278	caat|gt**G**cgtact	WK	23,792,275	23,792,586	IR
B2M	OTTHUMT00000 415957.1	15	45,008,525	tttttctccactgtcttttt cat**Gg**|atcga	DR	45,007,899	45,008,527	AA
FUS	OTTHUMT00000 433552.1	16	31,198,121	ctttcttctaacttgtcttc tcc**Gg**|cggaa	DR	31,196,500	31,198,123	IR
GOT2	OTTHUMT00000 258289.3	16	58,750,564	tatg|gt**G**agctac	NT	58,750,083	58,750,567	NA
EIF-4A1	OTTHUMT00000 226952.6	17	7,480,372	tattggcttttttttttcttc tct**Gg**|ccccc	DR	7,480,010	7,480,374	IR
C17orf76-AS1	OTTHUMT00000 130473.1	17	16,342,840	actgtcctgatgatacttgt aat**Gg**|gaagt	DR	16,342,728	16,342,842	IR
DDX5	OTTHUMT00000 444030.1	17	62,498,343	taacacaacttgtcttttaa ctt**Gg**|gtggc	DR	62,498,341	62,498,557	IR
SEPT9	OTTHUMT00000 436345.1	17	75,488,701	ccatctctctccctccttat ccc**Gg**|atcac	DR	75,486,941	75,488,703	NA
EIF3K	OTTHUMT00000 453406.1	19	39,125,631	cccttgctgcccctgtcacc tgc**Gg**|acagc	DR	39,123,318	39,125,633	NA
RPS19	OTTHUMT00000 463049.1	19	42,373,767	ctgcatgacccttccctccc cac**Gg**|cggcc	DR	42,373,284	42,373,769	NA
BAX	OTTHUMT00000 463049.1	19	42,373,826	acag|gt**G**aggcct	NT	42,373,823	42,375,419	NA
BAX	OTTHUMT00000 360763.1	19	49,458,803	cctcttcccttcctttctcc tct**Gg**|ggccc	DR	49,458,219	49,458,805	IR
RPL13A	OTTHUMT00000 258989.1	19	49,993,487	ctgctaagcctttccctccc tct**Gg**|gccgg	DR	49,993,179	49,993,489	IR
